# Atomization Energy Calculations in 13‐Atom Alkali Metal Clusters: Is There an Appropriate Exchange‐Correlation Functional?

**DOI:** 10.1002/jcc.70187

**Published:** 2025-07-21

**Authors:** Wagner F. D. Angelotti, Lucila C. Z. Angelotti, Roberto L. A. Haiduke

**Affiliations:** ^1^ Instituto de Ciências Tecnológicas e Exatas, Departamento de Matemática Aplicada Universidade Federal do Triângulo Mineiro Uberaba Brazil; ^2^ Centro Universitário Barão de Mauá São Paulo Brazil; ^3^ Departamento de Química e Física Molecular, Instituto de Química de São Carlos Universidade de São Paulo São Carlos Brazil

**Keywords:** alkali metal, atomization energy, clusters, electron correlation, exchange‐correlation functional

## Abstract

Density functional theory (DFT) is a treatment widely employed for exploring the electronic structure of atoms, molecules, solids, and complex systems. Despite its efficiency and popularity, the accuracy of DFT results is highly dependent on the choice of exchange–correlation (XC) functionals. This study evaluates several XC functionals for calculating atomization energies in 13‐atom homo‐ and heteronuclear alkali metal clusters (X_13_ and YX_12_, with X, Y = Li, Na, K, Rb, and Cs), comparing these results with reference data obtained from fixed‐node Diffusion Monte Carlo (DMC) simulations. Our findings emphasize the critical role of the correlation functional in achieving more accurate results. Moreover, empirical dispersion corrections are shown to be quite important for these systems. Notably, PBE and PBE0 functionals with D3‐BJ dispersion seem particularly reliable for atomization energy calculations in these clusters.

## Graphical abstract

Electronic correlation and dispersion corrections are crucial for accurately modeling the atomization energies of 13‐atom alkali‐metal clusters within DFT.

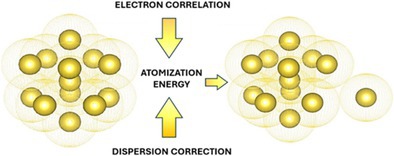

## 1 Introduction

Nanoparticles, characterized by their reduced dimensions, exhibit unique properties that have revolutionized diverse scientific and technological fields [1, 2]. Among these, metallic clusters—assemblies of metal atoms at the nanometer scale—stand out due to their exceptional electronic and chemical characteristics, making them promising candidates for a wide array of applications [3, 4]. The high reactivity and tunability of metallic clusters render them versatile applications spanning from catalysis to biomedicine [5, 6, 7].

Alkali metal clusters, in particular, offer unprecedented insights due to their relatively simple chemical interactions and the intricate relationship between size and composition [8, 9]. The accurate determination of atomization energies is crucial for understanding the stability and reactivity of these clusters since this knowledge can help in the discovery of novel applications. However, despite significant advances that have occurred, our understanding of these systems remains limited, especially in the case of clusters composed of the heaviest alkali metals (cesium and rubidium) [8] and some heteroclusters [9]. In order to contribute to this matter, a recent work comprising 13‐atom alkali metal systems (homo‐ and heteroatomic) was focused on providing more accurate atomization energy predictions by means of an advanced theoretical treatment [10]. Since experimental investigations on these systems are scarce and challenging, theoretical and computational approaches become essential for elucidating their structures [10, 11, 12, 13]. In this context, electronic structure methods, particularly Density Functional Theory (DFT) [14, 15], emerge as crucial tools for gaining a deeper understanding.

DFT is a widely used computational method for studying the electronic structure of complex systems by reducing the many‐electron problem to a single‐electron problem interacting with an effective potential [16, 17, 18, 19, 20]. Although DFT is powerful and versatile, the results provided are highly sensitive to factors such as the choice of exchange‐correlation (XC) functional and basis set [21, 22]. Therefore, the careful selection of these parameters is essential for achieving accurate results in specific chemical systems [23, 24]. While DFT has been particularly effective in investigating small alkali metal clusters, providing valuable insights into their structural, energetic, and spectroscopic properties [8, 25, 26, 27], studies of more complex systems such as homo‐ and heteronuclear 13‐atom alkali metal clusters remain relatively limited, and controversies persist [10]. Alternatively, the DFT investigations in larger systems such as metallic crystals have provided some clues regarding the performance of these treatments. Hence, PBE is normally highlighted among the best XC functionals in the description of cohesive energies of several metals, including those constituted by alkali, alkaline‐earth, and transition elements as well [28, 29, 30]. Anyway, similar accuracy investigations of atomization energies in small and medium‐size clusters are certainly desirable, and more XC categories can be easily included compared to solid‐state calculations.

In this work, we assess the performance of different XC functionals from DFT in providing atomization energies for homo‐ and heteronuclear 13‐atom alkali metal clusters based on icosahedral structures (X_13_ and YX_12_ (center‐ and face‐substituted), with X, Y = Li, Na, K, Rb, and Cs). The reference data to guide this comparison are retrieved from fixed‐node diffusion Monte Carlo (DMC) calculations previously reported by our group [10]. Notably, these DMC calculations are shown to be little sensible to finite‐size effects of the basis set used to obtain the trial wavefunctions once the quality of the nodes provided by these wavefunctions is the crucial aspect required to obtain accurate DMC energy estimates [31, 32, 33, 34]. In this case, although the DMC values are obtained from fixed nodes of the Kohn–Sham determinant achieved from previous UB3LYP/def2‐SVP(TNDF) calculations, they could be used as reliable benchmarks for comparison with electronic structure calculations performed using larger basis sets. Hence, through a detailed analysis of the results obtained, our study seeks to highlight the limitations of actual DFT approaches, trying to contribute to the development of more accurate computational methods for alkali metal clusters and related systems. Eventually, these findings can also guide the proposition of more successful treatments for general applications, which have direct implications for modeling metallic nanoparticles and alloys, ultimately benefiting the study of nanomaterials and catalytic processes in industrial applications.

## 2 Computational Methods

The equilibrium geometries of the alkali metal clusters reported in the Supporting Information of reference [10] are used for the electronic structure calculations done for 13‐atom systems. Hence, single‐point energy calculations were performed for these systems using a range of density functional approximations within the unrestricted DFT framework (UDFT). This range includes XC functionals from families such as local density approximation (LDA), generalized gradient approximation (GGA), meta‐GGAs, hybrids, meta‐GGA‐hybrids, range‐separated hybrids (RSHs), and double‐hybrids, as detailed in Table 1. The D3‐BJ empirical dispersion correction [69] was applied whenever available (see Table S1 for the corresponding parameters). The def2‐SVP and def2‐TZVP basis sets [70] combined with trail‐needs‐dirac‐Fock (TNDF) pseudopotentials (including scalar relativistic effects) [71, 72] are employed for the calculations. All computations were carried out using Gaussian 09 and 16 packages [73, 74], with default settings for the integration grid (Fine and Ultrafine grids, respectively) and integral accuracy criteria (10^−10^ and 10^−12^, respectively). In more detail, Gaussian 16 was considered for the calculations involving two double‐hybrid functionals not available in Gaussian 09 (PBE0DH and PBEQIDH).

**TABLE 1:** Summary of the exchange‐correlation functionals used in this study.

XC functional	Type	$E_{X}^{\mathrm{HF}}$	References
SVWN5	LDA	0	[35, 36]
BLYP	GGA	0	[37, 38]
BP86	GGA	0	[37, 39]
BPBE	GGA	0	[37, 40, 41]
HCTH	GGA	0	[42, 43, 44]
OLYP	GGA	0	[38, 45, 46]
PBE	GGA	0	[40, 41]
PW91	GGA	0	[47]
τ‐HCTH	META‐GGA	0	[48]
TPSS	META‐GGA	0	[49]
B3LYP	HYBRID	20	[37, 38, 50, 51]
B3P86	HYBRID	20	[37, 39, 50]
B3PW91	HYBRID	20	[37, 47, 50]
BHANDHLYP	HYBRID	50	[37, 38, 52]
MPW1PW91	HYBRID	25	[47, 53]
PBE0	HYBRID	25	[54]
X3LYP	HYBRID	21.8	[55]
BMK	META‐GGA‐HYBRID	42	[56]
M06‐2X	META‐GGA‐HYBRID	54	[57]
CAM‐B3LYP	RSH	19–65	[58]
CAM‐QTP‐00	RSH	54–91	[59]
CAM‐QTP‐01	RSH	23–100	[60]
CAM‐QTP‐02	RSH	28–100	[61]
LC‐BLYP	RSH	0–100	[62]
LC‐PBEPBE	RSH	0–100	[62]
LC‐ωPBE	RSH	0–100	[63]
LC‐QTP	RSH	0–100	[61]
ωB97X	RSH	16–100	[64]
B2PLYP	DOUBLE‐HYBRID	53	[65]
MPW2PLYP	DOUBLE‐HYBRID	55	[66]
PBE0DH	DOUBLE‐HYBRID	50	[67]
PBEQIDH	DOUBLE‐HYBRID	69	[68]

First, in order to perform a preliminary analysis, the next section employs the dendrogram for hierarchical clustering and the K‐means method for non‐hierarchical segmentation [75, 76], aiming to identify patterns and XC groups based on their similarities. The R software [77] was employed to generate both the hierarchical and non‐hierarchical clustering representations of the density functionals.

The electronic property evaluated in this work was the atomization energy (AE) of the clusters under study, which was obtained from the difference between the total electronic energy of the neutral cluster, *E* ^0^, and the sum of the electronic energies of the isolated atoms, *E*(*i*); that is,

(1) $$\mathrm{AE}=\left[\sum_{i=1}^{13}E\left(i\right)\right]-E^{0}$$

AEs are crucial quantities for understanding the structural stability of a cluster and can provide quite rigorous tests for the ability of electronic structure methods to describe electron correlation effects among other aspects [78].

Two additional tests were carried out with the calculation of single‐point energies in Gaussian 09. The first one extended the analysis of basis set effects and dispersion corrections to the 13‐atom clusters, using the def2‐QZVPPD [70, 79] basis set combined with D3‐BJ dispersion corrections, and the def2‐TZVP [70] basis set combined with D3 [80], D4 [81, 82] and VV10 [83, 84] dispersion corrections (the latter two are evaluated within the Orca 5.0.4 package [85, 86] considering def2/J auxiliary basis set [87]) for a few selected functionals (with the TNDF pseudopotentials). The second test examined the Li_4_ and Li_5_ clusters with def2‐TZVPP [70], def2‐QZVPP [70], cc‐pVTZ [88, 89, 90, 91], cc‐pVQZ [88, 89, 90, 91], aug‐cc‐pVTZ [88, 89, 90, 91], and aug‐cc‐pVQZ [88, 89, 90, 91] basis sets in an all‐electron approach.

## 3 Results and Discussion

### 3.1 Similarity Grouping of XC Functionals

**FIGURE 1:**
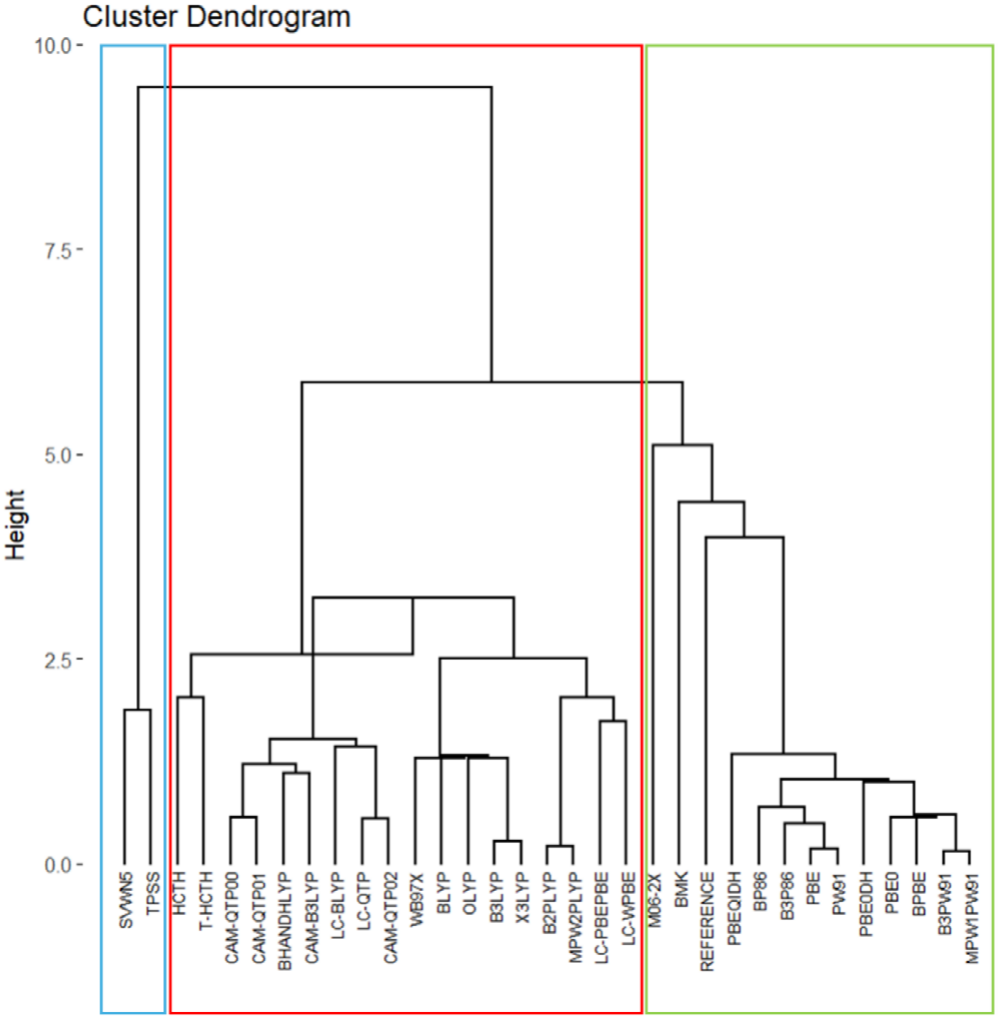
Dendrogram of exchange‐correlation functionals clustered based on their similarity to DMC calculations.

**FIGURE 2:**
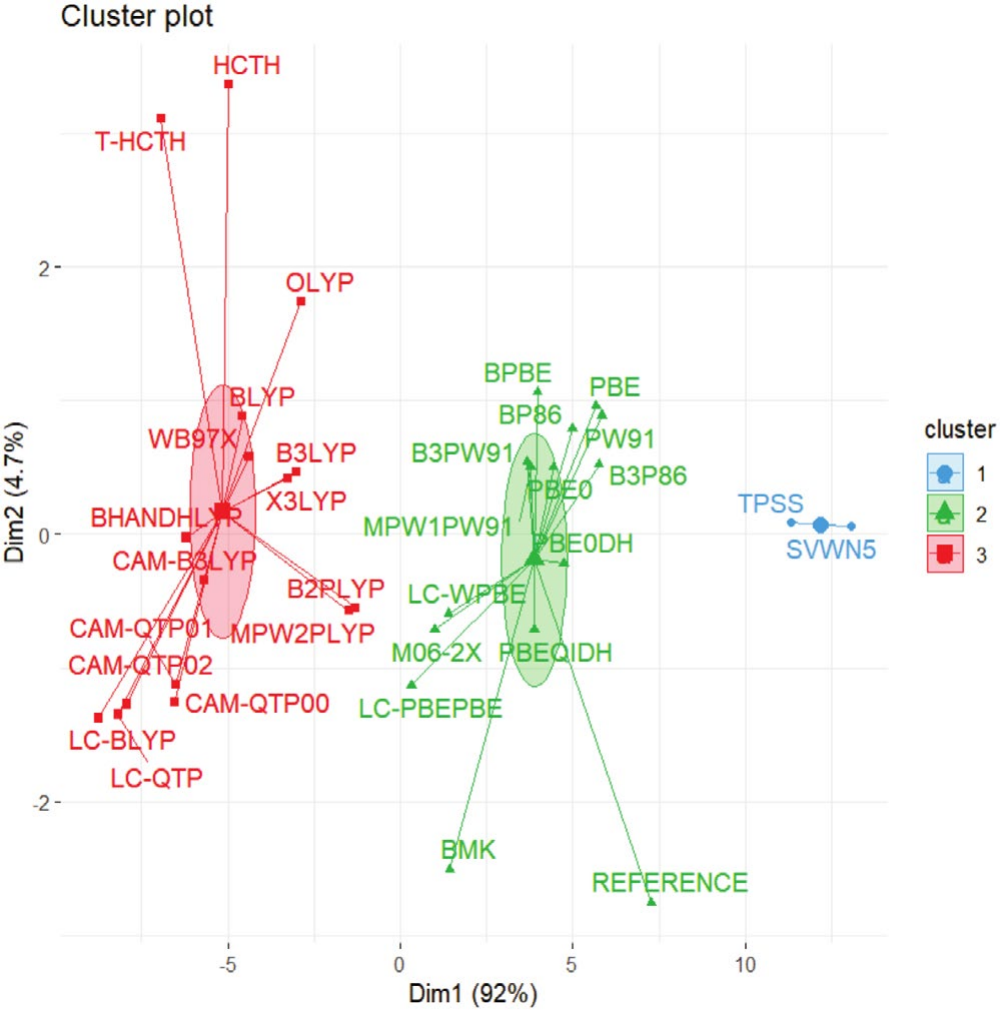
Exchange‐correlation functionals grouped by the K‐means method based on their similarity to DMC calculations.

Figures 1 and 2 present hierarchical and non‐hierarchical clustering analyses of the XC functionals used in this study (one LDA, seven GGAs, two meta‐GGAs, seven global hybrids, two meta‐GGA‐hybrids, nine RSHs, and four double‐hybrids), visualized respectively through a dendrogram and a scatter plot, which was generated using the K‐means method. These initial electronic structure calculations were performed with the def2‐SVP basis set and without empirical dispersion corrections, which seem adequate to provide a fast evaluation of several XC approximations with a lower demand for computational resources and mitigating self‐consistent field (SCF) convergence issues that tend to become more common as larger basis sets are considered. Details of the theoretical and practical framework underlying these clustering tools are provided in Data S1.

In these cases, as observed from the groupings achieved in Figures 1 and 2, the correlation functional appears to have a greater impact on the separation process than that from the exchange functional. This uncommon outcome can be understood considering the huge contribution provided by electron correlation corrections to these AE values. The comparison between results from unrestricted Hartree–Fock (UHF) calculations obtained with the largest basis set considered, def2‐TZVP, and DMC data indicates that electron correlation corrections respond for 55%–93% of these atomization energies, as shown in Table S1. As one can notice in the same table, exchange effects calculated at the UHF/def2‐TZVP are always less important, only exceeding 50% of total AEs in systems like NaLi_12_(f), RbLi_12_(f), CsLi_12_(f), RbK_12_(f), LiRb_12_(f), LiCs_12_(f), NaCs_12_(f), and RbCs_12_(f), that is, in face‐type substitutional clusters constituted by atoms presenting a more expressive difference in atomic sizes. Fixed‐node DMC is capable of including both static and dynamical electron correlation effects [10, 34, 92, 93, 94]. Hence, binding in alkali metal clusters is dominated by electron correlation effects. This finding seems quite consistent once these clusters are usually well described by jellium models [95, 96]. Certainly, this aspect poses a challenge to modern DFT treatments, as one can notice by the significant deviations provided by many XC functionals.

Hence, Figures 1 and 2 categorize the XC functionals into three major groups. First, SVWN and TPSS constitute a group quite discrepant from DMC values (Group 1). Next, LYP‐based correlation functionals are all grouped together (Group 3), also displaying small similarity with the reference values. Other functionals like HCTH, τ‐HCTH, and ωB97X also end up grouped together with LYP‐based functionals.

Density functionals clustered in Group 2 of Figure 2 demonstrate a closer match to the reference data, as also evidenced by the lower Mean Absolute Deviations (MADs) shown in Table S2. This group is composed mainly of functionals that include PBE‐, PW91‐, and P86‐type correlation expressions. Moreover, other functionals like BMK and M06‐2X are also classified as members of this group, although their similarity seems smaller compared to the majority of other XC functionals within this group.

### 3.2 Dispersion and Basis Set Size Effects

Given the dominant influence of electron correlation on these AE values and recognizing that van der Waals interactions comprise both attractive (London dispersion and other terms) and repulsive components, dispersion corrections that account for the London forces can provide important contributions in this context. Moreover, as the basis set initially considered is small (def2‐SVP), it is important to evaluate the role of larger sets as well. Consequently, we extended our study by employing the def2‐TZVP basis set and incorporating dispersion effects (D3‐BJ approach; see Table S3 for the parameters) to gain a more comprehensive understanding of atomization energies in such materials. This approach allows a better assessment of the potential usage of each XC functional for future research and technological application investigations involving alkali metal clusters and related systems.

The selection of XC functionals for this subsequent investigation was guided by both the prior analysis and the availability of empirical dispersion corrections (D3‐BJ). Thus, the BP86, BPBE, PBE, B3PW91, PBE0, and LC‐ωPBE functionals were chosen based on K‐means analysis and the dendrogram. These members include three GGAs, two hybrids, and one RSH. Other functionals like PW91, B3P86, MPW1PW91, PBE0DH, and PBEQIDH also seem quite similar to the ones selected but are not considered at this stage once the D3‐BJ correction is not available for them within the computational package used. Additionally, B3LYP (a widely used hybrid functional), TPSS (a meta‐GGA functional), and B2PLYP (a double‐hybrid functional) were also included for a more complete investigation bearing other XC categories as well, resulting in a total of nine XC functionals.

**FIGURE 3:**
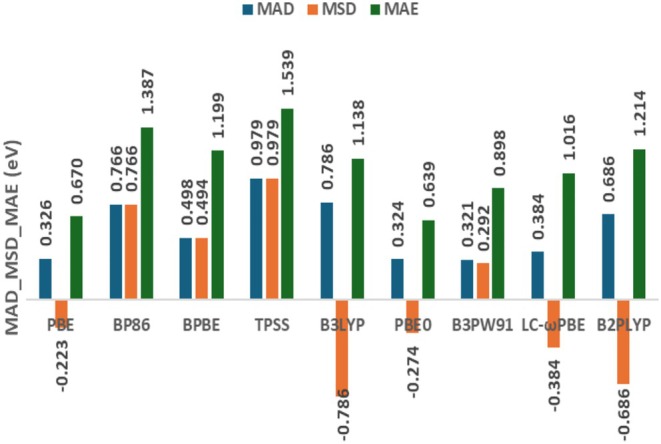
Mean absolute deviation (MAD), mean signed deviation (MSD), and maximum absolute error (MAE) values obtained for the nine density functionals selected by using the def2‐TZVP basis set and D3‐BJ dispersion corrections (in eV).

The MAD and Mean Signed Deviation (MSD) values, as shown in Figure 3 and Table S4, indicate that the PBE, PBE0, and B3PW91 functionals are the most accurate options for the systems studied, as combined with the D3‐BJ dispersion correction (MAD = 0.321–0.326 eV and MSD between −0.274 and 0.292 eV). However, the Maximum Absolute Error (MAE), also illustrated in Figure 3, points to advantages of PBE and PBE0 (0.670 and 0.639 eV) compared to B3PW91 (0.898 eV). Although the correlation functional chosen seems to be the most important factor to obtain more accurate AE values for these alkali metal clusters, the proper balance between exchange and correlation functionals has some effect on the XC performance as well, as noted by comparing PBE with BPBE. Hence, the combination of PBE for exchange and correlation seems more successful here. The three functionals included to increase the XC diversity (TPSS, B3LYP, and B2PLYP) are among the worst of the nine members considered at this stage, together with BP86.

In addition to MAD and MSD, the standard deviation of signed errors for the AEs calculated (SD[MSD]) is also evaluated, as shown in Table S4. A closer examination of SD(MSD) reveals a noteworthy trend following a progression along Jacob's Ladder of DFT (GGA >META‐GGA >HYBRID > RSH). The only exception is B3PW91, which shows an SD(MSD) similar to that from GGAs. The double hybrid considered, B2PLYP, exhibits an SD(MSD) result almost equal to that from TPSS. Smaller SD(MSD) values suggest that some functionals tend to provide similar deviations along all systems, that is, with some more consistent systematic trend for overestimation or underestimation of AEs. Perhaps, this systematic trend can be mitigated depending on future reparameterizations of dispersion corrections. Anyway, the difference between the SD(MSD) values among the nine XC functionals considered is not so significant, varying from 0.211 to 0.374 eV. Another standard deviation metric is obtained in terms of absolute errors, SD (MAD), which is also available in Table S4. In this case, PBE and PBE0 are clearly superior compared to the other functionals investigated at this stage, providing absolute deviations that tend to show smaller variability along the systems investigated.

**FIGURE 4:**
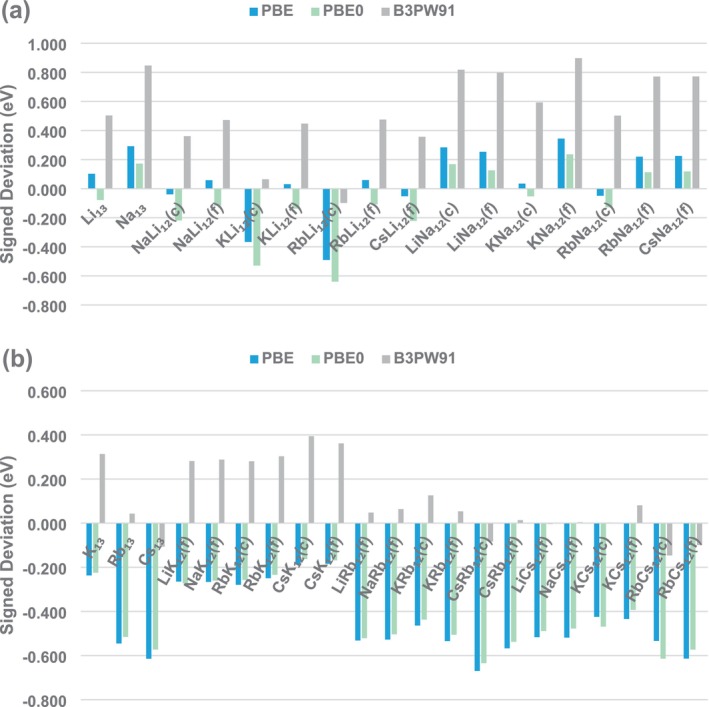
Signed deviations in atomization energies for 13‐atom homo‐ and heteronuclear alkali metal clusters: (a) XY_12_ (X = Li, Na, K, Rb, and Cs; Y = Li and Na); and (b) XY_12_ (X = Li, Na, K, Rb, and Cs; Y = K, Rb, and Cs), as obtained by using the PBE, PBE0, and B3PW91 functionals with the def2‐TZVP basis set and D3‐BJ dispersion corrections compared to DMC reference data (in eV).

Another important factor to highlight, as shown in Figure 4, is that among the three most successful functionals, PBE and PBE0 exhibit a consistent trend of systematically underestimating the AE values for clusters majorly composed of heavier alkali metals (K, Rb, and Cs), providing much better agreement for DE values in clusters constituted mainly by lithium and sodium. This seems easy to understand once our previous work found evidence that the need for a multi‐reference wavefunction treatment increases as the atomic number of the alkali metal considered becomes larger and that the static correlation effects reinforce the binding in such clusters [10]. Hence, one could expect that DFT treatments will tend to provide smaller AE values in such cases, considering the difficulties in dealing with static correlation effects. In contrast, the B3PW91 functional shows a trend not so easy to interpret, with systematically overestimated AE values for clusters majorly composed of lighter elements (Li, Na, and K) and much better accordance for clusters constituted mainly by rubidium and cesium. The excellent performance of PBE (GGA) and PBE0 (global hybrid) is also in agreement with the findings of Kostko and coworkers, who demonstrated the suitability of PBE for describing structural and energetic properties of sodium clusters [97, 98].

Interestingly, our results show that several higher‐level functionals do not outperform simpler GGAs and traditional hybrids for describing binding in alkali metal clusters. This behavior may be attributed to a combination of factors: (i) overparameterization and limited transferability of these functionals, which are often fitted to *p*‐block molecular datasets; (ii) intrinsic challenges in describing metallic systems, where electron delocalization is critical; and (iii) inadequate treatment of static correlation. These findings reinforce that functional complexity does not necessarily guarantee higher accuracy and highlight the need for more robust and transferable XC functionals for metallic systems.

Thus, considering the results obtained in this study and the limitations discussed, the PBE and PBE0 functionals (combined with D3‐BJ corrections) are recommended to describe the atomization energies in alkali metal clusters.

Next, evaluating the dispersion effects in more detail, as shown in Table S5, all the nine functionals considered at this stage indicate that this factor increases the AE values in alkali metal clusters by 0.43–1.86 eV. Among them, PBE always provides the smallest dispersion corrections in each system (from 0.43 to 0.68 eV), followed closely by PBE0 (between 0.54 and 0.75 eV). On the other hand, the largest dispersion corrections are those given by BPBE (for systems mainly composed by Li and Na) and BP86 (for the remaining ones). For comparison, B3PW91 provides much larger dispersion corrections than PBE and PBE0, from 1.12 to 1.59 eV. Hence, the curious behavior of B3PW91 deviations with respect to DMC along the systems mentioned earlier in this work seems to be a direct consequence of too large dispersion corrections included for this XC functional. Hence, a delicate tuning of empirical dispersion contributions also appears to be an important factor for obtaining accurate AE values for these alkali metal clusters. Dispersion effects have been shown to be essential for accurately predicting both the putative global minimum structures of sodium clusters (10–20 atoms) and the alkali metal lattice constants [99, 100].

Indeed, investigating the basis set size effect, as shown in Table S6, all the nine XC functionals considered here agree that the AE values decrease between −0.34 and −1.35 eV when moving from def2‐SVP to def2‐TZVP sets for systems primarily formed of the lightest alkali metals (Li, Na, and K). On the other hand, the AE results for clusters mainly constituted by rubidium and cesium are much less sensitive to such basis set effects.

Next, in order to further extend our analysis, we performed additional calculations using the def2‐QZVPPD basis set for the PBE and PBE0 functionals combined with the D3‐BJ dispersion correction. The results, as shown in Table S7, indicate that the MADs and MSDs obtained with this larger basis set do not show a significant improvement compared to those from the def2‐TZVP. Thus, the MAD and MSD values for PBE are 0.361 eV and −0.289 eV, respectively, while the corresponding values for PBE0 are 0.341 and −0.324 eV. These results suggest that the basis set size increment from def2‐TZVP to def2‐QZVPPD has a minimal impact on the accuracy of the predicted AEs, reinforcing the favorable balance between accuracy and computational cost already achieved with the triple‐zeta basis set. The additional basis set increment effect is almost negligible for clusters mainly composed of K, Rb, and Cs. However, this same basis set augmentation tends to increase slightly the AEs from clusters mainly composed of lithium (from 0.12 to 0.25 eV), while the values obtained for clusters predominantly constituted by sodium still decrease (by −0.37 to −0.60 eV).

Additionally, we investigated the performance of the original D3 expression without BJ damping (see Table S3), D4, and VV10 dispersion corrections in combination with the PBE functional and employing the def2‐TZVP basis set (see Table S8). The results indicate that the simplest D3 correction yields slightly lower deviations (MAD = 0.298 eV, MSD = −0.196 eV) compared to the D3‐BJ correction (MAD = 0.326 eV, MSD = −0.223 eV). These differences are relatively small, suggesting that both corrections exhibit similar overall performance in our study. In addition, our analysis reveals that the PBE‐D3(BJ) scheme performs better than D4 and VV10 in terms of both MAD (0.444 and 0.351 eV, respectively) and MSD (−0.441 and −0.330 eV, respectively). Thus, the transferability and reliability of commonly used dispersion corrections to metallic systems may be limited, as their molecular‐based parameterization might not accurately capture the intricate electronic characteristics of metallic bonding. Anyway, future investigation is required to address this point more carefully.

### 3.3 Final Remarks on the Performance of PBE: Predicting Atomization Energies in Small Lithium Clusters With Different Structural Arrangements

Since PBE combined with the D3‐BJ dispersion correction is quite successful for predicting AE values in fixed icosahedral‐based structures of the 13‐atom alkali metal clusters investigated here, a final performance evaluation would require considering different geometries of the same clusters for assessing the XC functional ability for properly quantifying the binding in each nuclear arrangement. This would be crucial for global minimum search investigations. Hence, this combination (PBE‐D3‐BJ) was used for predicting the AE results of small lithium clusters in alternative structures and spin‐multiplicity states, Li_4_ (*sym*‐linear, linear, and rhomboid geometries in singlet and triple states) and Li_5_ (linear, W, and trigonal bipyramid arrangements in doublet and quartet states), by using different basis sets in an all‐electron approach. This protocol was followed because a recent benchmark dataset was provided by Kermani and Truhlar for such species [101], being used as a reference here. The best AE estimates recommended in that work were calculated by the W3X‐L method, which is a composite procedure including post‐CCSD(T) corrections, being capable of dealing with moderate multi‐reference character [102]. The geometries considered at this point were retrieved from the reference work.

**FIGURE 5:**
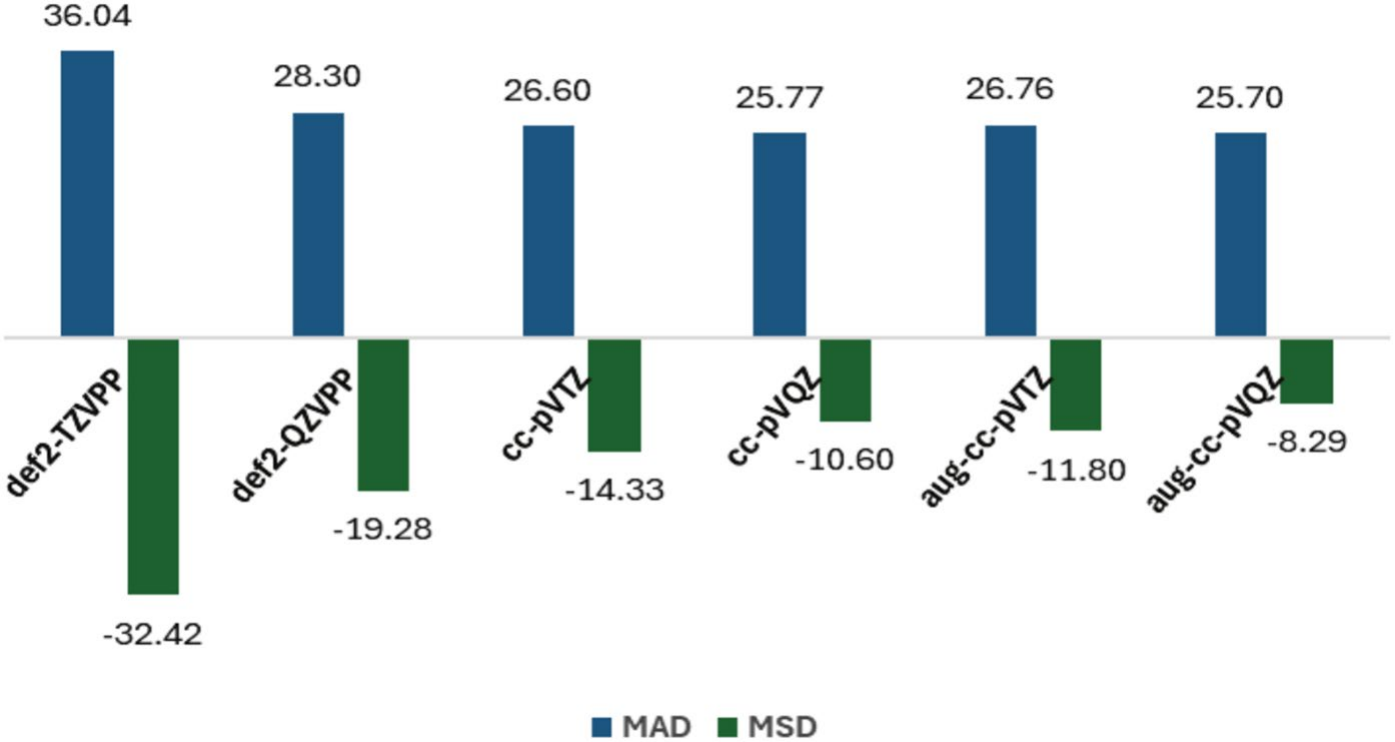
Mean absolute deviation (MAD) and mean signed deviation (MSD) in atomization energy values per atom calculated with PBE‐D3‐BJ and different basis sets for Li_4_ and Li_5_ clusters (in meV).

The MAD and MSD values regarding the AE results per atom with respect to W3X‐L [101], as shown in Figure 5 and Table S9, indicate that the PBE functional combined with the D3‐BJ dispersion correction and the largest basis sets provide quite accurate AE results for this dataset, including 12 systems. It is worth noticing that the MAD obtained for 13‐atom clusters with PBE‐D3‐BJ and the def2‐TZVP basis set (0.326 eV) refers to the total deviation per cluster, whereas the MADs presented for the smallest lithium clusters investigated here are reported on a per atom metric. Thus, when properly normalized, the MAD for 13‐atom clusters is approximately 25 meV/atom, which is similar to the values obtained for Li_4_ and Li_5_.

Thus, the PBE functional with D3‐BJ corrections (MAD and MSD of 26 and *−*8 meV, respectively, with the aug‐cc‐pVQZ basis set) exhibits a competitive performance when compared to the best XC functional investigated in reference [94], revM06‐L (MAD and MSD of 16 and *−*6 meV, respectively, with the cc‐pCVQZ basis set), surpassing all the other XC approaches considered there. In addition, PBE with D3‐BJ dispersion is more accurate than the G4 composite approach and several wavefunction methods also evaluated in reference [101], such as CCSD, MP2, MP3, MP4D, MP4DQ, and MP4SDQ. More importantly, comparing the PBE/cc‐pVTZ performance also evaluated in that study with our PBE‐D3‐BJ/cc‐pVTZ results, one can notice that dispersion corrections are important to improve the MADs (from 34 to 27 meV) and, mainly, the MSDs (from *−*32 to *−*14 meV). In other words, dispersion corrections mitigate the PBE trend for underestimating binding for such systems.

Finally, there are other studies reporting that FN‐DMC results based on single‐reference trial wavefunctions can be accurately used as reference data for pure lithium clusters of varying sizes, such as the investigations carried out by Brito et al. [103, 104, 105]. In one of these studies, PBE was also shown to be the best XC functional considered to describe binding energies from Li_2_ to Li_8_ [105]. Hence, this last work also reinforces the general conclusions drawn here.

## 4 Conclusions

This study evaluates various XC functionals for obtaining the atomization energy of 13‐atom homo‐ and heteronuclear alkali metal clusters. Our findings underscore the critical importance of choosing an appropriate correlation functional for an accurate description of binding in such clusters. Moreover, dispersion corrections also seem quite important for reliable predictions of these AE values. Hence, functionals such as PBE and PBE0 provided some of the best results when combined with the D3‐BJ empirical dispersion treatment, highlighting their potential as reliable tools for binding predictions in related materials. Hence, the dispersion corrections for PBE and PBE0 provide AE results larger by 0.43–0.75 eV for these clusters. In this aspect, the B3PW91 functional combined with the D3‐BJ correction seems to overestimate the atomization energies in general cases, which may be caused by too large dispersion effects. The combination of PBE with D3‐BJ was also quite accurate for evaluating the AE results per atom of smaller lithium clusters (Li_4_ and Li_5_) in different structures and spin multiplicity states, providing a reliable option for global minimum search investigations in similar systems.

Finally, this work reinforces that the binding description in alkali metal clusters constitutes a challenge to modern DFT approaches due to the preponderance of electron correlation effects (static and dynamical), composing an interesting test set to evaluate new functionals under development. Hence, this study provides a complement to DFT investigations of metallic crystals, which indicate that PBE can render accurate cohesive energies as well. Thus, PBE should remain reliable along a wide system size scale, which would provide important insights on cohesive energy trends along growth processes of metallic nanoparticles, for instance. Moreover, considering that heteronuclear alkali metal clusters were evaluated here as well, PBE could be recommended for metal doping and nanoalloy studies. In addition, although PBE0 also furnishes competitive results for the clusters investigated, the limitations of solid‐state calculations restrict the applications of global hybrids in such cases. Thus, our results provide relevant insights for designing computational strategies for material science studies aiming at technological applications, which certainly will rely on more accurate and efficient descriptions of energy changes in several processes.

Future research should broaden the scope of functional evaluation to include a wider range of cluster sizes, compositions, and electronic properties. Our study provides a sound foundation for these future investigations and highlights the potential of DFT methods in addressing challenging nanoscale phenomena.

## Conflicts of Interest

The authors declare no conflicts of interest.

## Supporting information


**Data S1.** Supporting Information.

## Data Availability

The data that supports the findings of this study are available in the Supporting Information of this article.
