# Supplementary material for: Atomization Energy Calculations in 13‐Atom Alkali Metal Clusters: Is There an Appropriate Exchange‐Correlation Functional?
Source: J Comput Chem. 2025 Jul 21;46(20):e70187. doi: 10.1002/jcc.70187 (PMC12277945; doi:10.1002/jcc.70187)
Supplement: Supplementary file 1 — Data S1. Supporting Information. [file JCC-46-0-s001.zip › SI_data_X13_DFT_rev-combinado.pdf]

Table S1. Atomization energies (DE, in eV) and the respective percentage contributions from exchange and electronic correlation effects, as determined by Diffusion M[illegible]

* Diffusion Monte Carlo (DMC) data from reference *Ch*[illegible]

Systems	Unrestricted Hartree-Fock method Basis Set DE(def2-SVP)	DE(def2-TZVP)	DMC* DE(REFERENCE)	%Corr. def2-SVP	%Corr. def2-TZVP
Li_{13}	6.189	5.800	12.937	52.2	55.2
Na_{13}	3.134	2.511	8.954	65.0	72.0
K_{13}	1.811	1.460	7.048	74.3	79.3
Rb_{13}	0.915	0.952	6.662	86.3	85.7
Cs_{13}	0.703	0.752	6.056	88.4	87.6
$NaLi_{12}$(c)	5.159	4.687	11.978	56.9	60.9
$NaLi_{12}$(f)	6.083	5.649	12.669	52.0	55.4
KLi_{12}(c)	3.877	3.468	10.99	64.7	68.4
KLi_{12}(f)	5.949	5.439	12.493	52.4	56.5
$RbLi_{12}$(c)	3.659	2.963	10.389	64.8	71.5
$RbLi_{12}$(f)	5.877	5.496	12.398	52.6	55.7
$CsLi_{12}$(f)	5.863	5.498	12.496	53.1	56.0
$LiNa_{12}$(c)	3.726	3.017	9.313	60.0	67.6
$LiNa_{12}$(f)	3.353	2.705	9.208	63.6	70.6
KNa_{12}(c)	2.358	1.858	8.433	72.0	78.0
KNa_{12}(f)	3.085	2.480	8.709	64.6	71.5
$RbNa_{12}$(c)	2.313	1.573	8.207	71.8	80.8
$RbNa_{12}$(f)	2.938	2.450	8.775	66.5	72.1
$CsNa_{12}$(f)	2.933	2.451	8.739	66.4	72.0
LiK_{12}(f)	2.113	1.795	7.379	71.4	75.7
NaK_{12}(f)	2.019	1.684	7.227	72.1	76.7
RbK_{12}(c)	1.756	1.294	6.969	74.8	81.4
RbK_{12}(f)	1.820	1.543	7.004	74.0	78.0
CsK_{12}(c)	1.537	1.042	6.761	77.3	84.6

Systems	Basis Set DE_Exch(def2-TZVP)	DMC* DE(REFER[illegible]
Li_{13}	5.802	12.937
Na_{13}	2.419	8.954
K_{13}	1.552	7.048
Rb_{13}	2.165	6.662
Cs_{13}	2.220	6.056
$NaLi_{12}$(c)	3.893	11.978
$NaLi_{12}$(f)	6.528	12.669
KLi_{12}(c)	2.779	10.99
KLi_{12}(f)	5.813	12.493
$RbLi_{12}$(c)	1.542	10.389
$RbLi_{12}$(f)	6.457	12.398
$CsLi_{12}$(f)	6.488	12.496
$LiNa_{12}$(c)	3.349	9.313
$LiNa_{12}$(f)	3.210	9.208
KNa_{12}(c)	0.982	8.433
KNa_{12}(f)	2.888	8.709
$RbNa_{12}$(c)	0.653	8.207
$RbNa_{12}$(f)	2.829	8.775
$CsNa_{12}$(f)	2.862	8.739
LiK_{12}(f)	3.464	7.379
NaK_{12}(f)	3.100	7.227
RbK_{12}(c)	1.225	6.969
RbK_{12}(f)	5.096	7.004
CsK_{12}(c)	0.914	6.761

CsK_{12}(f)	1.785	1.442	6.896	74.1	79.1
$LiRb_{12}$(f)	1.077	1.461	6.958	84.5	79.0
$NaRb_{12}$(f)	1.255	1.008	6.839	81.6	85.3
KRb_{12}(c)	1.155	1.162	6.699	82.8	82.6
KRb_{12}(f)	1.195	0.982	6.703	82.2	85.3
$CsRb_{12}$(c)	0.694	0.729	6.673	89.6	89.1
$CsRb_{12}$(f)	0.916	0.954	6.642	86.2	85.6
$LiCs_{12}$(f)	1.347	1.420	6.324	78.7	77.5
$NaCs_{12}$(f)	1.198	1.331	6.225	80.8	78.6
KCs_{12}(c)	0.513	0.501	6.007	91.5	91.7
KCs_{12}(f)	1.025	0.767	5.983	82.9	87.2
$RbCs_{12}$(c)	0.320	0.416	6.042	94.7	93.1
$RbCs_{12}$(f)	0.698	1.051	6.111	88.6	82.8

CsK_{12}(f)	2.026	6.896
$LiRb_{12}$(f)	4.130	6.958
$NaRb_{12}$(f)	2.668	6.839
KRb_{12}(c)	2.561	6.699
KRb_{12}(f)	2.172	6.703
$CsRb_{12}$(c)	1.493	6.673
$CsRb_{12}$(f)	2.323	6.642
$LiCs_{12}$(f)	4.513	6.324
$NaCs_{12}$(f)	4.355	6.225
KCs_{12}(c)	1.147	6.007
KCs_{12}(f)	2.123	5.983
$RbCs_{12}$(c)	1.042	6.042
$RbCs_{12}$(f)	4.024	6.111

	**def2-SVP**	**def2-TZVP**
**Mean**	72.8	75.9
**Minimun**	52.0	55.2
**Maximun**	94.7	93.1

**Exch(def2-TZVP) (in Hartree)**	
Li	-0.117
Na	-0.106
K	-0.086
Rb	-0.081
Cs	-0.074

lonte Carlo.

em. Phys. **2023**, *565* , 111767.

%Exch. def2-TZVP		Exch(def2-TZVP) (in Hartree)
44.8		-1.733
27.0		-1.465
22.0		-1.178
32.5		-1.135
36.7		-1.043
32.5		-1.652
51.5		-1.749
25.3		-1.591
46.5		-1.703
14.8		-1.541
52.1		-1.721
51.9		-1.715
36.0		-1.510
34.9		-1.505
11.6		-1.393
33.2		-1.463
8.0		-1.376
32.2		-1.456
32.7		-1.450
46.9		-1.279
42.9		-1.255
17.6		-1.161
72.8		-1.303
13.5		-1.142

29.4	-1.183
59.4	-1.243
39.0	-1.178
38.2	-1.154
32.4	-1.140
22.4	-1.103
35.0	-1.133
71.4	-1.170
70.0	-1.153
19.1	-1.016
35.5	-1.052
17.2	-1.007
65.8	-1.116

	**def2-TZVP**
**Mean**	36.6
**Minimun**	8.0
**Maximun**	72.8

Table S2. Mean Absolute Deviation (MAD) and Mean Signed Deviation (MSD) of dissociation energies (eV), calculated with the def2svp basis set and without empirical

	Hartree-Fock method	Density Functional					
		LSDA	GGA				
**Systems**	**UHF**	**SVWN5**	**PBE**	**BP86**	**BLYP**	**BPBE**	**PW91**
Li_{13}	6.189	14.553	13.408	13.260	11.436	13.122	13.422
Na_{13}	3.134	10.566	9.431	9.257	7.612	9.134	9.454
K_{13}	1.811	8.026	7.008	6.817	5.288	6.757	7.017
Rb_{13}	0.915	6.848	5.553	5.509	4.069	5.279	5.591
Cs_{13}	0.703	6.199	4.962	4.914	3.564	4.696	5.000
$NaLi_{12}$(c)	5.159	13.633	12.416	12.297	10.566	12.120	12.431
$NaLi_{12}$(f)	6.083	14.236	13.095	12.945	11.133	12.808	13.110
KLi_{12}(c)	3.877	12.371	11.061	11.006	9.380	10.771	11.077
KLi_{12}(f)	5.949	14.034	12.901	12.755	10.954	12.617	12.918
$RbLi_{12}$(c)	3.659	12.029	10.672	10.625	8.998	10.388	10.674
$RbLi_{12}$(f)	5.877	13.864	12.703	12.565	10.771	12.416	12.721
$CsLi_{12}$(f)	5.863	13.843	12.689	12.552	10.765	12.402	12.708
$LiNa_{12}$(c)	3.726	10.998	9.893	9.694	7.997	9.600	9.917
$LiNa_{12}$(f)	3.353	10.819	9.676	9.511	7.860	9.381	9.700
KNa_{12}(c)	2.358	9.802	8.597	8.452	6.872	8.302	8.617
KNa_{12}(f)	3.085	10.379	9.257	9.080	7.442	8.964	9.280
$RbNa_{12}$(c)	2.313	9.846	8.604	8.492	6.933	8.330	8.623
$RbNa_{12}$(f)	2.938	10.211	9.065	8.891	7.256	8.767	9.089
$CsNa_{12}$(f)	2.933	10.176	9.038	8.864	7.235	8.741	9.062
LiK_{12}(f)	2.113	8.365	7.317	7.153	5.632	7.065	7.331
NaK_{12}(f)	2.019	8.199	7.167	6.987	5.461	6.915	7.179
RbK_{12}(c)	1.756	8.047	6.987	6.835	5.369	6.734	7.007
RbK_{12}(f)	1.820	8.013	6.979	6.801	5.287	6.728	6.992
CsK_{12}(c)	1.537	8.034	6.937	6.817	5.375	6.692	6.958

CsK_{12}(f)	1.785	7.929	6.900	6.719	5.206	6.649	6.912
$LiRb_{12}$(f)	1.077	7.243	5.938	5.903	4.458	5.664	5.976
$NaRb_{12}$(f)	1.255	7.081	5.790	5.740	4.292	5.515	5.826
KRb_{12}(c)	1.155	7.077	5.816	5.766	4.329	5.546	5.852
KRb_{12}(f)	1.195	6.982	5.708	5.656	4.214	5.437	5.744
$CsRb_{12}$(c)	0.694	6.781	5.455	5.435	4.028	5.180	5.493
$CsRb_{12}$(f)	0.916	6.805	5.520	5.474	4.040	5.248	5.557
$LiCs_{12}$(f)	1.347	6.621	5.374	5.344	3.996	5.111	5.415
$NaCs_{12}$(f)	1.198	6.484	5.253	5.207	3.851	4.990	5.293
KCs_{12}(c)	0.513	6.398	5.243	5.158	3.777	4.986	5.279
KCs_{12}(f)	1.025	6.364	5.140	5.085	3.727	4.875	5.176
$RbCs_{12}$(c)	0.320	6.204	5.007	4.930	3.542	4.744	5.044
$RbCs_{12}$(f)	0.698	6.257	5.014	4.970	3.617	4.749	5.053

**Absolute deviations**							
	**Hartree-Fock method**	**Density Functional**					
		**LSDA**	**GGA**				
**Systems**	**UHF**	**SVWN5**	**PBE**	**BP86**	**BLYP**	**BPBE**	**PW91**
Li_{13}	6.748	1.616	0.471	0.323	1.501	0.185	0.485
Na_{13}	5.820	1.612	0.477	0.303	1.342	0.180	0.500
K_{13}	5.237	0.978	0.040	0.231	1.760	0.291	0.031
Rb_{13}	5.747	0.186	1.109	1.153	2.593	1.383	1.071
Cs_{13}	5.353	0.143	1.094	1.142	2.492	1.360	1.056
$NaLi_{12}$(c)	6.819	1.655	0.438	0.319	1.412	0.142	0.453
$NaLi_{12}$(f)	6.586	1.567	0.426	0.276	1.536	0.139	0.441
KLi_{12}(c)	7.113	1.381	0.071	0.016	1.610	0.219	0.087
KLi_{12}(f)	6.544	1.541	0.408	0.262	1.539	0.124	0.425

$RbLi_{12}$(c)	6.730	1.640	0.283	0.236	1.391	0.001	0.285
$RbLi_{12}$(f)	6.521	1.466	0.305	0.167	1.627	0.018	0.323
$CsLi_{12}$(f)	6.633	1.347	0.193	0.056	1.731	0.094	0.212
$LiNa_{12}$(c)	5.587	1.685	0.580	0.381	1.316	0.287	0.604
$LiNa_{12}$(f)	5.855	1.611	0.468	0.303	1.348	0.173	0.492
KNa_{12}(c)	6.075	1.369	0.164	0.019	1.561	0.131	0.184
KNa_{12}(f)	5.624	1.670	0.548	0.371	1.267	0.255	0.571
$RbNa_{12}$(c)	5.894	1.639	0.397	0.285	1.274	0.123	0.416
$RbNa_{12}$(f)	5.837	1.436	0.290	0.116	1.519	0.008	0.314
$CsNa_{12}$(f)	5.806	1.437	0.299	0.125	1.504	0.002	0.323
LiK_{12}(f)	5.266	0.986	0.062	0.226	1.747	0.314	0.048
NaK_{12}(f)	5.208	0.972	0.060	0.240	1.766	0.312	0.048
RbK_{12}(c)	5.213	1.078	0.018	0.134	1.600	0.235	0.038
RbK_{12}(f)	5.184	1.009	0.025	0.203	1.717	0.276	0.012
CsK_{12}(c)	5.224	1.273	0.176	0.056	1.386	0.069	0.197
CsK_{12}(f)	5.111	1.033	0.004	0.177	1.690	0.247	0.016
$LiRb_{12}$(f)	5.881	0.285	1.020	1.055	2.500	1.294	0.982
$NaRb_{12}$(f)	5.584	0.242	1.049	1.099	2.547	1.324	1.013
KRb_{12}(c)	5.544	0.378	0.883	0.933	2.370	1.153	0.847
KRb_{12}(f)	5.508	0.279	0.995	1.047	2.489	1.266	0.959
$CsRb_{12}$(c)	5.979	0.108	1.218	1.238	2.645	1.493	1.180
$CsRb_{12}$(f)	5.726	0.163	1.122	1.168	2.602	1.394	1.085
$LiCs_{12}$(f)	4.977	0.297	0.950	0.980	2.328	1.213	0.909
$NaCs_{12}$(f)	5.027	0.259	0.972	1.018	2.374	1.235	0.932
KCs_{12}(c)	5.494	0.391	0.764	0.849	2.230	1.021	0.728
KCs_{12}(f)	4.958	0.381	0.843	0.898	2.256	1.108	0.807
$RbCs_{12}$(c)	5.722	0.162	1.035	1.112	2.500	1.298	0.998
$RbCs_{12}$(f)	5.413	0.146	1.097	1.141	2.494	1.362	1.058

	Hartree-Fock method	Density Functional					
		LSDA	GGA				
	UHF	SVWN5	PBE	BP86	BLYP	BPBE	PW91
**MAD**	5.772	0.957	0.550	0.531	1.880	0.587	0.544

Signed deviations

	Hartree-Fock method	Density Functional					
		LSDA	GGA				
**Systems**	UHF	SVWN5	PBE	BP86	BLYP	BPBE	PW91
Li_{13}	-6.748	1.616	0.471	0.323	-1.501	0.185	0.485
Na_{13}	-5.820	1.612	0.477	0.303	-1.342	0.180	0.500
K_{13}	-5.237	0.978	-0.040	-0.231	-1.760	-0.291	-0.031
Rb_{13}	-5.747	0.186	-1.109	-1.153	-2.593	-1.383	-1.071
Cs_{13}	-5.353	0.143	-1.094	-1.142	-2.492	-1.360	-1.056
$NaLi_{12}$(c)	-6.819	1.655	0.438	0.319	-1.412	0.142	0.453
$NaLi_{12}$(f)	-6.586	1.567	0.426	0.276	-1.536	0.139	0.441
KLi_{12}(c)	-7.113	1.381	0.071	0.016	-1.610	-0.219	0.087
KLi_{12}(f)	-6.544	1.541	0.408	0.262	-1.539	0.124	0.425
$RbLi_{12}$(c)	-6.730	1.640	0.283	0.236	-1.391	-0.001	0.285
$RbLi_{12}$(f)	-6.521	1.466	0.305	0.167	-1.627	0.018	0.323
$CsLi_{12}$(f)	-6.633	1.347	0.193	0.056	-1.731	-0.094	0.212
$LiNa_{12}$(c)	-5.587	1.685	0.580	0.381	-1.316	0.287	0.604
$LiNa_{12}$(f)	-5.855	1.611	0.468	0.303	-1.348	0.173	0.492
KNa_{12}(c)	-6.075	1.369	0.164	0.019	-1.561	-0.131	0.184

KNa_{12}(f)	-5.624	1.670	0.548	0.371	-1.267	0.255	0.571
$RbNa_{12}$(c)	-5.894	1.639	0.397	0.285	-1.274	0.123	0.416
$RbNa_{12}$(f)	-5.837	1.436	0.290	0.116	-1.519	-0.008	0.314
$CsNa_{12}$(f)	-5.806	1.437	0.299	0.125	-1.504	0.002	0.323
LiK_{12}(f)	-5.266	0.986	-0.062	-0.226	-1.747	-0.314	-0.048
NaK_{12}(f)	-5.208	0.972	-0.060	-0.240	-1.766	-0.312	-0.048
RbK_{12}(c)	-5.213	1.078	0.018	-0.134	-1.600	-0.235	0.038
RbK_{12}(f)	-5.184	1.009	-0.025	-0.203	-1.717	-0.276	-0.012
CsK_{12}(c)	-5.224	1.273	0.176	0.056	-1.386	-0.069	0.197
CsK_{12}(f)	-5.111	1.033	0.004	-0.177	-1.690	-0.247	0.016
$LiRb_{12}$(f)	-5.881	0.285	-1.020	-1.055	-2.500	-1.294	-0.982
$NaRb_{12}$(f)	-5.584	0.242	-1.049	-1.099	-2.547	-1.324	-1.013
KRb_{12}(c)	-5.544	0.378	-0.883	-0.933	-2.370	-1.153	-0.847
KRb_{12}(f)	-5.508	0.279	-0.995	-1.047	-2.489	-1.266	-0.959
$CsRb_{12}$(c)	-5.979	0.108	-1.218	-1.238	-2.645	-1.493	-1.180
$CsRb_{12}$(f)	-5.726	0.163	-1.122	-1.168	-2.602	-1.394	-1.085
$LiCs_{12}$(f)	-4.977	0.297	-0.950	-0.980	-2.328	-1.213	-0.909
$NaCs_{12}$(f)	-5.027	0.259	-0.972	-1.018	-2.374	-1.235	-0.932
KCs_{12}(c)	-5.494	0.391	-0.764	-0.849	-2.230	-1.021	-0.728
KCs_{12}(f)	-4.958	0.381	-0.843	-0.898	-2.256	-1.108	-0.807
$RbCs_{12}$(c)	-5.722	0.162	-1.035	-1.112	-2.500	-1.298	-0.998
$RbCs_{12}$(f)	-5.413	0.146	-1.097	-1.141	-2.494	-1.362	-1.058

	**Hartree-Fock method**	**Density Functional**					
		**LSDA**	**GGA**				
	**UHF**	**SVWN5**	**PBE**	**BP86**	**BLYP**	**BPBE**	**PW91**
**MSD**	-5.772	0.957	-0.225	-0.336	-1.880	-0.499	-0.200

l dispersion correction, using various density functionals, compared to DMC reference data.

		META-GGA		HYBRID GGA			
OLYP	HCTH	TPSS	τ-HCTH	B3LYP	PBE0	B3P86	B3PW91
12.067	12.142	14.183	11.563	11.604	13.028	13.327	12.903
8.009	7.866	10.291	7.552	7.833	9.162	9.356	9.017
5.553	5.388	7.962	5.192	5.555	6.836	6.951	6.696
4.198	3.591	6.514	3.288	4.395	5.470	5.681	5.352
3.641	2.965	5.903	2.715	3.879	4.886	5.089	4.775
11.132	11.103	13.171	10.580	10.728	12.037	12.344	11.924
11.738	11.783	13.881	11.233	11.307	12.726	13.017	12.598
9.877	9.740	11.835	9.230	9.549	10.701	11.069	10.622
11.533	11.568	13.689	11.038	11.126	12.532	12.829	12.407
9.435	9.274	11.485	8.789	9.172	10.330	10.667	10.249
11.334	11.317	13.493	10.794	10.954	12.349	12.644	12.222
11.323	11.316	13.476	10.792	10.943	12.332	12.632	12.205
8.427	8.326	10.780	7.958	8.219	9.628	9.797	9.470
8.264	8.119	10.528	7.802	8.075	9.397	9.606	9.256
7.242	6.989	9.478	6.649	7.122	8.371	8.570	8.235
7.826	7.682	10.123	7.373	7.663	8.993	9.180	8.848
7.272	6.896	9.503	6.609	7.173	8.368	8.605	8.261
7.636	7.460	9.929	7.134	7.487	8.813	8.993	8.664
7.609	7.427	9.901	7.102	7.463	8.785	8.968	8.637
5.873	5.667	8.259	5.483	5.882	7.124	7.278	6.995
5.717	5.531	8.120	5.327	5.725	6.990	7.121	6.854
5.583	5.335	7.944	5.176	5.613	6.799	6.952	6.677
5.536	5.329	7.929	5.123	5.551	6.809	6.933	6.673
5.576	5.230	7.899	5.083	5.625	6.754	6.953	6.653

5.452	5.249	7.848	5.050	5.470	6.732	6.851	6.594
4.589	3.976	6.888	3.688	4.767	5.830	6.063	5.719
4.433	3.846	6.746	3.536	4.613	5.697	5.910	5.579
4.447	3.900	6.758	3.577	4.642	5.723	5.935	5.605
4.346	3.766	6.669	3.474	4.533	5.617	5.825	5.499
4.150	3.511	6.427	3.161	4.356	5.376	5.614	5.270
4.163	3.555	6.479	3.257	4.364	5.437	5.647	5.319
4.059	3.383	6.318	3.151	4.288	5.270	5.507	5.169
3.928	3.273	6.205	3.016	4.159	5.167	5.382	5.058
3.858	3.357	6.160	3.077	3.951	5.035	5.199	4.921
3.814	3.166	6.079	2.919	4.036	5.053	5.256	4.943
3.627	3.036	5.940	2.786	3.741	4.816	4.981	4.706
3.694	3.018	5.958	2.762	3.932	4.937	5.146	4.828

		META-GGA		HYBRID GGA			
OLYP	HCTH	TPSS	τ-HCTH	B3LYP	PBE0	B3P86	B3PW91
0.870	0.795	1.246	1.374	1.333	0.091	0.390	0.034
0.945	1.088	1.337	1.402	1.121	0.208	0.402	0.063
1.495	1.660	0.914	1.856	1.493	0.212	0.097	0.352
2.464	3.071	0.148	3.374	2.267	1.192	0.981	1.310
2.415	3.091	0.153	3.341	2.177	1.170	0.967	1.281
0.846	0.875	1.193	1.398	1.250	0.059	0.366	0.054
0.931	0.886	1.212	1.436	1.362	0.057	0.348	0.071
1.113	1.250	0.845	1.760	1.441	0.289	0.079	0.368
0.960	0.925	1.196	1.455	1.367	0.039	0.336	0.086

0.954	1.115	1.096	1.600	1.217	0.059	0.278	0.140
1.064	1.081	1.095	1.604	1.444	0.049	0.246	0.176
1.173	1.180	0.980	1.704	1.553	0.164	0.136	0.291
0.886	0.987	1.467	1.355	1.094	0.315	0.484	0.157
0.944	1.089	1.320	1.406	1.133	0.189	0.398	0.048
1.191	1.444	1.045	1.784	1.311	0.062	0.137	0.198
0.883	1.027	1.414	1.336	1.046	0.284	0.471	0.139
0.935	1.311	1.296	1.598	1.034	0.161	0.398	0.054
1.139	1.315	1.154	1.641	1.288	0.038	0.218	0.111
1.130	1.312	1.162	1.637	1.276	0.046	0.229	0.102
1.506	1.712	0.880	1.896	1.497	0.255	0.101	0.384
1.510	1.696	0.893	1.900	1.502	0.237	0.106	0.373
1.386	1.634	0.975	1.793	1.356	0.170	0.017	0.292
1.468	1.675	0.925	1.881	1.453	0.195	0.071	0.331
1.185	1.531	1.138	1.678	1.136	0.007	0.192	0.108
1.444	1.647	0.952	1.846	1.426	0.164	0.045	0.302
2.369	2.982	0.070	3.270	2.191	1.128	0.895	1.239
2.406	2.993	0.093	3.303	2.226	1.142	0.929	1.260
2.252	2.799	0.059	3.122	2.057	0.976	0.764	1.094
2.357	2.937	0.034	3.229	2.170	1.086	0.878	1.204
2.523	3.162	0.246	3.512	2.317	1.297	1.059	1.403
2.479	3.087	0.163	3.385	2.278	1.205	0.995	1.323
2.265	2.941	0.006	3.173	2.036	1.054	0.817	1.155
2.297	2.952	0.020	3.209	2.066	1.058	0.843	1.167
2.149	2.650	0.153	2.930	2.056	0.972	0.808	1.086
2.169	2.817	0.096	3.064	1.947	0.930	0.727	1.040
2.415	3.006	0.102	3.256	2.301	1.226	1.061	1.336
2.417	3.093	0.153	3.349	2.179	1.174	0.965	1.283

		META-GGA		HYBRID GGA			
**OLYP**	**HCTH**	**TPSS**	**τ-HCTH**	**B3LYP**	**PBE0**	**B3P86**	**B3PW91**
1.593	1.914	0.736	2.239	1.633	0.512	0.493	0.579

		META-GGA		HYBRID GGA			
**OLYP**	**HCTH**	**TPSS**	**τ-HCTH**	**B3LYP**	**PBE0**	**B3P86**	**B3PW91**
-0.870	-0.795	1.246	-1.374	-1.333	0.091	0.390	-0.034
-0.945	-1.088	1.337	-1.402	-1.121	0.208	0.402	0.063
-1.495	-1.660	0.914	-1.856	-1.493	-0.212	-0.097	-0.352
-2.464	-3.071	-0.148	-3.374	-2.267	-1.192	-0.981	-1.310
-2.415	-3.091	-0.153	-3.341	-2.177	-1.170	-0.967	-1.281
-0.846	-0.875	1.193	-1.398	-1.250	0.059	0.366	-0.054
-0.931	-0.886	1.212	-1.436	-1.362	0.057	0.348	-0.071
-1.113	-1.250	0.845	-1.760	-1.441	-0.289	0.079	-0.368
-0.960	-0.925	1.196	-1.455	-1.367	0.039	0.336	-0.086
-0.954	-1.115	1.096	-1.600	-1.217	-0.059	0.278	-0.140
-1.064	-1.081	1.095	-1.604	-1.444	-0.049	0.246	-0.176
-1.173	-1.180	0.980	-1.704	-1.553	-0.164	0.136	-0.291
-0.886	-0.987	1.467	-1.355	-1.094	0.315	0.484	0.157
-0.944	-1.089	1.320	-1.406	-1.133	0.189	0.398	0.048
-1.191	-1.444	1.045	-1.784	-1.311	-0.062	0.137	-0.198

-0.883	-1.027	1.414	-1.336	-1.046	0.284	0.471	0.139
-0.935	-1.311	1.296	-1.598	-1.034	0.161	0.398	0.054
-1.139	-1.315	1.154	-1.641	-1.288	0.038	0.218	-0.111
-1.130	-1.312	1.162	-1.637	-1.276	0.046	0.229	-0.102
-1.506	-1.712	0.880	-1.896	-1.497	-0.255	-0.101	-0.384
-1.510	-1.696	0.893	-1.900	-1.502	-0.237	-0.106	-0.373
-1.386	-1.634	0.975	-1.793	-1.356	-0.170	-0.017	-0.292
-1.468	-1.675	0.925	-1.881	-1.453	-0.195	-0.071	-0.331
-1.185	-1.531	1.138	-1.678	-1.136	-0.007	0.192	-0.108
-1.444	-1.647	0.952	-1.846	-1.426	-0.164	-0.045	-0.302
-2.369	-2.982	-0.070	-3.270	-2.191	-1.128	-0.895	-1.239
-2.406	-2.993	-0.093	-3.303	-2.226	-1.142	-0.929	-1.260
-2.252	-2.799	0.059	-3.122	-2.057	-0.976	-0.764	-1.094
-2.357	-2.937	-0.034	-3.229	-2.170	-1.086	-0.878	-1.204
-2.523	-3.162	-0.246	-3.512	-2.317	-1.297	-1.059	-1.403
-2.479	-3.087	-0.163	-3.385	-2.278	-1.205	-0.995	-1.323
-2.265	-2.941	-0.006	-3.173	-2.036	-1.054	-0.817	-1.155
-2.297	-2.952	-0.020	-3.209	-2.066	-1.058	-0.843	-1.167
-2.149	-2.650	0.153	-2.930	-2.056	-0.972	-0.808	-1.086
-2.169	-2.817	0.096	-3.064	-1.947	-0.930	-0.727	-1.040
-2.415	-3.006	-0.102	-3.256	-2.301	-1.226	-1.061	-1.336
-2.417	-3.093	-0.153	-3.349	-2.179	-1.174	-0.965	-1.283

		META-GGA		HYBRID GGA			
**OLYP**	**HCTH**	**TPSS**	**τ-HCTH**	**B3LYP**	**PBE0**	**B3P86**	**B3PW91**
-1.593	-1.914	0.672	-2.239	-1.633	-0.432	-0.217	-0.554

			HYBRID META-GGA		RANGE-SEPARATED GGA	
MPW1PW91	X3LYP	BHANDHLYP	M06-2X	BMK	LC-BLYP	LC-PBEPBE
12.909	11.537	10.826	12.316	10.868	9.784	11.458
9.047	7.787	7.221	8.182	7.936	6.396	7.955
6.729	5.510	5.073	6.220	6.821	4.451	5.947
5.375	4.368	4.016	3.857	5.024	3.549	4.846
4.796	3.851	3.522	5.812	4.264	3.484	4.694
11.916	10.667	9.975	11.293	9.542	8.779	11.089
12.608	11.241	10.552	11.998	11.610	9.552	11.264
10.583	9.487	8.821	10.388	8.792	8.414	9.815
12.416	11.060	10.370	11.888	11.472	9.475	11.095
10.205	9.112	8.473	9.899	8.426	8.086	9.491
12.233	10.891	10.228	11.621	11.325	9.400	10.997
12.215	10.879	10.210	11.788	10.773	9.380	10.975
9.514	8.172	7.598	8.725	9.097	7.291	8.951
9.282	8.027	7.451	8.434	8.894	7.152	8.689
8.255	7.081	6.568	7.847	7.159	5.388	7.780
8.879	7.618	7.058	8.040	8.445	6.760	8.316
8.259	7.127	6.603	7.793	7.740	6.343	7.760
8.698	7.444	6.898	7.776	8.301	6.618	8.164
8.671	7.420	6.873	7.940	8.286	6.592	8.136
7.018	5.835	5.375	6.530	7.156	5.206	6.217
6.884	5.680	5.242	6.368	6.995	5.103	6.552
6.698	5.570	5.118	6.154	6.741	4.470	5.858
6.704	5.507	5.070	6.095	6.698	4.467	5.942
6.656	5.578	5.124	6.460	6.879	4.289	6.274

6.627	5.427	4.994	6.200	6.773	4.443	5.941
5.734	4.736	4.359	4.299	5.840	4.313	5.570
5.600	4.584	4.225	4.157	5.619	4.209	5.481
5.628	4.611	4.243	4.088	5.246	3.752	5.090
5.521	4.504	4.145	4.077	5.626	3.638	4.993
5.281	4.328	3.982	4.139	5.013	3.933	5.156
5.342	4.336	3.986	3.997	5.411	3.549	4.837
5.183	4.257	3.912	6.061	5.548	3.943	5.104
5.079	4.130	3.804	5.948	5.374	3.856	5.037
4.937	3.920	3.539	5.687	4.822	3.446	4.735
4.963	4.006	3.666	5.866	5.152	3.614	4.835
4.717	3.710	3.346	5.416	5.082	3.276	4.546
4.848	3.904	3.574	5.678	5.076	3.528	4.734

			**HYBRID META-GGA**		**RANGE-SEPARATED GGA**	
**MPW1PW91**	**X3LYP**	**BHANDHLYP**	**M06-2X**	**BMK**	**LC-BLYP**	**LC-PBEPBE**
0.028	1.400	2.111	0.621	2.069	3.153	1.479
0.093	1.167	1.733	0.772	1.018	2.558	0.999
0.319	1.538	1.975	0.828	0.227	2.597	1.101
1.287	2.294	2.646	2.805	1.638	3.113	1.816
1.260	2.205	2.534	0.244	1.792	2.572	1.362
0.062	1.311	2.003	0.685	2.436	3.199	0.889
0.061	1.428	2.117	0.671	1.059	3.117	1.405
0.407	1.503	2.169	0.602	2.198	2.576	1.175
0.077	1.433	2.123	0.605	1.021	3.018	1.398

0.184	1.277	1.916	0.490	1.963	2.303	0.898
0.165	1.507	2.170	0.777	1.073	2.998	1.401
0.281	1.617	2.286	0.708	1.723	3.116	1.521
0.201	1.141	1.715	0.588	0.216	2.022	0.362
0.074	1.181	1.757	0.774	0.314	2.056	0.519
0.178	1.352	1.865	0.586	1.274	3.045	0.653
0.170	1.091	1.651	0.669	0.264	1.949	0.393
0.052	1.080	1.604	0.414	0.467	1.864	0.447
0.077	1.331	1.877	0.999	0.474	2.157	0.611
0.068	1.319	1.866	0.799	0.453	2.147	0.603
0.361	1.544	2.004	0.849	0.223	2.173	1.162
0.343	1.547	1.985	0.859	0.232	2.124	0.675
0.271	1.399	1.851	0.815	0.228	2.499	1.111
0.300	1.497	1.934	0.909	0.306	2.537	1.062
0.105	1.183	1.637	0.301	0.118	2.472	0.487
0.269	1.469	1.902	0.696	0.123	2.453	0.955
1.224	2.222	2.599	2.659	1.118	2.645	1.388
1.239	2.255	2.614	2.682	1.220	2.630	1.358
1.071	2.088	2.456	2.611	1.453	2.947	1.609
1.182	2.199	2.558	2.626	1.077	3.065	1.710
1.392	2.345	2.691	2.534	1.660	2.740	1.517
1.300	2.306	2.656	2.645	1.231	3.093	1.805
1.141	2.067	2.412	0.263	0.776	2.381	1.220
1.146	2.095	2.421	0.277	0.851	2.369	1.188
1.070	2.087	2.468	0.320	1.185	2.561	1.272
1.020	1.977	2.317	0.117	0.831	2.369	1.148
1.325	2.332	2.696	0.626	0.960	2.766	1.496
1.263	2.207	2.537	0.433	1.035	2.583	1.377

			HYBRID META-GGA		RANGE-SEPARATED GGA	
**MPW1PW91**	**X3LYP**	**BHANDHLYP**	**M06-2X**	**BMK**	**LC-BLYP**	**LC-PBEPBE**
0.569	1.676	2.158	0.996	0.981	2.594	1.124

			HYBRID META-GGA		RANGE-SEPARATED GGA	
**MPW1PW91**	**X3LYP**	**BHANDHLYP**	**M06-2X**	**BMK**	**LC-BLYP**	**LC-PBEPBE**
-0.028	-1.400	-2.111	-0.621	-2.069	-3.153	-1.479
0.093	-1.167	-1.733	-0.772	-1.018	-2.558	-0.999
-0.319	-1.538	-1.975	-0.828	-0.227	-2.597	-1.101
-1.287	-2.294	-2.646	-2.805	-1.638	-3.113	-1.816
-1.260	-2.205	-2.534	-0.244	-1.792	-2.572	-1.362
-0.062	-1.311	-2.003	-0.685	-2.436	-3.199	-0.889
-0.061	-1.428	-2.117	-0.671	-1.059	-3.117	-1.405
-0.407	-1.503	-2.169	-0.602	-2.198	-2.576	-1.175
-0.077	-1.433	-2.123	-0.605	-1.021	-3.018	-1.398
-0.184	-1.277	-1.916	-0.490	-1.963	-2.303	-0.898
-0.165	-1.507	-2.170	-0.777	-1.073	-2.998	-1.401
-0.281	-1.617	-2.286	-0.708	-1.723	-3.116	-1.521
0.201	-1.141	-1.715	-0.588	-0.216	-2.022	-0.362
0.074	-1.181	-1.757	-0.774	-0.314	-2.056	-0.519
-0.178	-1.352	-1.865	-0.586	-1.274	-3.045	-0.653

0.170	-1.091	-1.651	-0.669	-0.264	-1.949	-0.393
0.052	-1.080	-1.604	-0.414	-0.467	-1.864	-0.447
-0.077	-1.331	-1.877	-0.999	-0.474	-2.157	-0.611
-0.068	-1.319	-1.866	-0.799	-0.453	-2.147	-0.603
-0.361	-1.544	-2.004	-0.849	-0.223	-2.173	-1.162
-0.343	-1.547	-1.985	-0.859	-0.232	-2.124	-0.675
-0.271	-1.399	-1.851	-0.815	-0.228	-2.499	-1.111
-0.300	-1.497	-1.934	-0.909	-0.306	-2.537	-1.062
-0.105	-1.183	-1.637	-0.301	0.118	-2.472	-0.487
-0.269	-1.469	-1.902	-0.696	-0.123	-2.453	-0.955
-1.224	-2.222	-2.599	-2.659	-1.118	-2.645	-1.388
-1.239	-2.255	-2.614	-2.682	-1.220	-2.630	-1.358
-1.071	-2.088	-2.456	-2.611	-1.453	-2.947	-1.609
-1.182	-2.199	-2.558	-2.626	-1.077	-3.065	-1.710
-1.392	-2.345	-2.691	-2.534	-1.660	-2.740	-1.517
-1.300	-2.306	-2.656	-2.645	-1.231	-3.093	-1.805
-1.141	-2.067	-2.412	-0.263	-0.776	-2.381	-1.220
-1.146	-2.095	-2.421	-0.277	-0.851	-2.369	-1.188
-1.070	-2.087	-2.468	-0.320	-1.185	-2.561	-1.272
-1.020	-1.977	-2.317	-0.117	-0.831	-2.369	-1.148
-1.325	-2.332	-2.696	-0.626	-0.960	-2.766	-1.496
-1.263	-2.207	-2.537	-0.433	-1.035	-2.583	-1.377

			**HYBRID META-GGA**		**RANGE-SEPARATED GGA**	
**MPW1PW91**	**X3LYP**	**BHANDHLYP**	**M06-2X**	**BMK**	**LC-BLYP**	**LC-PBEPBE**
-0.537	-1.676	-2.158	-0.996	-0.975	-2.594	-1.124

RANGE-SEPARATED HYBRID GGA

CAM-B3LYP	LC-ωPBE	ωB97X	LC-QTP	CAM-QTP00	CAM-QTP01	CAM-QTP02
10.973	12.134	11.348	9.769	10.136	10.122	9.861
7.350	8.469	7.764	6.394	6.703	6.663	6.461
4.814	6.369	5.324	4.451	4.697	4.631	4.468
4.181	5.218	4.221	3.549	3.737	3.747	3.557
3.689	4.674	3.380	3.484	3.684	3.679	3.487
10.108	11.120	10.506	8.808	9.157	9.848	8.887
10.710	11.908	11.057	9.550	9.918	9.959	9.703
8.966	9.840	9.404	8.411	8.738	8.731	8.477
10.064	11.704	10.345	9.472	9.800	9.797	9.541
8.608	9.500	9.035	8.084	8.394	8.386	8.145
9.946	11.585	10.190	9.397	9.716	9.716	9.461
10.376	11.549	10.186	9.377	9.699	9.698	9.443
7.734	8.956	8.126	7.290	7.578	7.561	7.331
7.582	8.693	7.985	7.151	7.431	7.415	7.187
6.710	7.772	7.172	6.329	6.593	6.580	6.363
7.187	8.315	7.594	6.759	7.040	7.018	6.794
6.744	7.754	7.116	6.342	6.615	6.600	6.378
7.030	8.162	7.414	6.617	6.892	6.872	6.651
7.006	8.134	7.378	6.591	6.867	6.848	6.626
5.516	6.632	5.522	5.205	5.441	5.429	5.219
4.988	6.539	5.827	5.103	5.331	5.321	5.114
4.848	6.293	5.182	4.913	5.155	5.131	4.928
4.822	6.349	5.261	4.893	5.133	5.116	4.912
5.263	6.263	5.598	4.921	5.170	5.147	4.938

5.132	6.280	5.564	4.822	5.057	5.036	4.835
4.523	5.551	4.547	4.313	4.513	4.516	4.317
4.393	5.461	4.447	4.209	4.402	4.407	4.211
4.403	5.446	4.120	4.160	4.376	4.364	4.167
4.309	5.356	4.366	3.638	4.303	4.302	4.105
4.151	5.136	4.197	3.933	4.142	4.135	3.939
4.152	5.198	4.182	3.549	3.762	3.748	3.558
4.094	5.087	4.023	3.943	4.126	4.140	3.942
3.989	5.019	3.952	3.856	4.034	4.050	3.854
3.687	4.712	3.767	3.436	3.643	3.630	3.443
3.830	4.816	3.526	3.614	3.818	3.811	3.618
3.502	4.521	3.538	3.266	3.467	3.457	3.272
3.747	4.715	3.725	3.528	3.727	3.724	3.531

RANGE-SEPARATED HYBRID GGA

CAM-B3LYP	LC-ωPBE	ωB97X	LC-QTP	CAM-QTP00	CAM-QTP01	CAM-QTP02
1.964	0.803	1.589	3.168	2.801	2.815	3.076
1.604	0.485	1.190	2.560	2.251	2.291	2.493
2.234	0.679	1.724	2.597	2.351	2.417	2.580
2.481	1.444	2.441	3.113	2.925	2.915	3.105
2.367	1.382	2.676	2.572	2.372	2.377	2.569
1.870	0.858	1.472	3.170	2.821	2.130	3.091
1.959	0.761	1.612	3.119	2.751	2.710	2.966
2.024	1.150	1.586	2.579	2.252	2.259	2.513
2.429	0.789	2.148	3.021	2.693	2.696	2.952

1.781	0.889	1.354	2.305	1.995	2.003	2.244
2.452	0.813	2.208	3.001	2.682	2.682	2.937
2.120	0.947	2.310	3.119	2.797	2.798	3.053
1.579	0.357	1.187	2.023	1.735	1.752	1.982
1.626	0.515	1.223	2.057	1.777	1.793	2.021
1.723	0.661	1.261	2.104	1.840	1.853	2.070
1.522	0.394	1.115	1.950	1.669	1.691	1.915
1.463	0.453	1.091	1.865	1.592	1.607	1.829
1.745	0.613	1.361	2.158	1.883	1.903	2.124
1.733	0.605	1.361	2.148	1.872	1.891	2.113
1.863	0.747	1.857	2.174	1.938	1.950	2.160
2.239	0.688	1.400	2.124	1.896	1.906	2.113
2.121	0.676	1.787	2.056	1.814	1.838	2.041
2.182	0.655	1.743	2.111	1.871	1.888	2.092
1.498	0.498	1.163	1.840	1.591	1.614	1.823
1.764	0.616	1.332	2.074	1.839	1.860	2.061
2.435	1.407	2.411	2.645	2.445	2.442	2.641
2.446	1.378	2.392	2.630	2.437	2.432	2.628
2.296	1.253	2.579	2.539	2.323	2.335	2.532
2.394	1.347	2.337	3.065	2.400	2.401	2.598
2.522	1.537	2.476	2.740	2.531	2.538	2.734
2.490	1.444	2.460	3.093	2.880	2.894	3.084
2.230	1.237	2.301	2.381	2.198	2.184	2.382
2.236	1.206	2.273	2.369	2.191	2.175	2.371
2.320	1.295	2.240	2.571	2.364	2.377	2.564
2.153	1.167	2.457	2.369	2.165	2.172	2.365
2.540	1.521	2.504	2.776	2.575	2.585	2.770
2.364	1.396	2.386	2.583	2.384	2.387	2.580

RANGE-SEPARATED HYBRID GGA						
CAM-B3LYP	LC-ωPBE	ωB97X	LC-QTP	CAM-QTP00	CAM-QTP01	CAM-QTP02
2.075	0.937	1.865	2.507	2.240	2.231	2.464

RANGE-SEPARATED HYBRID GGA						
CAM-B3LYP	LC-ωPBE	ωB97X	LC-QTP	CAM-QTP00	CAM-QTP01	CAM-QTP02
-1.964	-0.803	-1.589	-3.168	-2.801	-2.815	-3.076
-1.604	-0.485	-1.190	-2.560	-2.251	-2.291	-2.493
-2.234	-0.679	-1.724	-2.597	-2.351	-2.417	-2.580
-2.481	-1.444	-2.441	-3.113	-2.925	-2.915	-3.105
-2.367	-1.382	-2.676	-2.572	-2.372	-2.377	-2.569
-1.870	-0.858	-1.472	-3.170	-2.821	-2.130	-3.091
-1.959	-0.761	-1.612	-3.119	-2.751	-2.710	-2.966
-2.024	-1.150	-1.586	-2.579	-2.252	-2.259	-2.513
-2.429	-0.789	-2.148	-3.021	-2.693	-2.696	-2.952
-1.781	-0.889	-1.354	-2.305	-1.995	-2.003	-2.244
-2.452	-0.813	-2.208	-3.001	-2.682	-2.682	-2.937
-2.120	-0.947	-2.310	-3.119	-2.797	-2.798	-3.053
-1.579	-0.357	-1.187	-2.023	-1.735	-1.752	-1.982
-1.626	-0.515	-1.223	-2.057	-1.777	-1.793	-2.021
-1.723	-0.661	-1.261	-2.104	-1.840	-1.853	-2.070

-1.522	-0.394	-1.115	-1.950	-1.669	-1.691	-1.915
-1.463	-0.453	-1.091	-1.865	-1.592	-1.607	-1.829
-1.745	-0.613	-1.361	-2.158	-1.883	-1.903	-2.124
-1.733	-0.605	-1.361	-2.148	-1.872	-1.891	-2.113
-1.863	-0.747	-1.857	-2.174	-1.938	-1.950	-2.160
-2.239	-0.688	-1.400	-2.124	-1.896	-1.906	-2.113
-2.121	-0.676	-1.787	-2.056	-1.814	-1.838	-2.041
-2.182	-0.655	-1.743	-2.111	-1.871	-1.888	-2.092
-1.498	-0.498	-1.163	-1.840	-1.591	-1.614	-1.823
-1.764	-0.616	-1.332	-2.074	-1.839	-1.860	-2.061
-2.435	-1.407	-2.411	-2.645	-2.445	-2.442	-2.641
-2.446	-1.378	-2.392	-2.630	-2.437	-2.432	-2.628
-2.296	-1.253	-2.579	-2.539	-2.323	-2.335	-2.532
-2.394	-1.347	-2.337	-3.065	-2.400	-2.401	-2.598
-2.522	-1.537	-2.476	-2.740	-2.531	-2.538	-2.734
-2.490	-1.444	-2.460	-3.093	-2.880	-2.894	-3.084
-2.230	-1.237	-2.301	-2.381	-2.198	-2.184	-2.382
-2.236	-1.206	-2.273	-2.369	-2.191	-2.175	-2.371
-2.320	-1.295	-2.240	-2.571	-2.364	-2.377	-2.564
-2.153	-1.167	-2.457	-2.369	-2.165	-2.172	-2.365
-2.540	-1.521	-2.504	-2.776	-2.575	-2.585	-2.770
-2.364	-1.396	-2.386	-2.583	-2.384	-2.387	-2.580

RANGE-SEPARATED HYBRID GGA

**CAM-B3LYP**	**LC-ωPBE**	**ωB97X**	**LC-QTP**	**CAM-QTP00**	**CAM-QTP01**	**CAM-QTP02**
-2.075	-0.937	-1.865	-2.507	-2.240	-2.231	-2.464

DOUBLE HYBRID GGA				DMC*
B2PLYP	MPW2PLYP	PBEQIDH	PBE0DH	REFERENCE
11.522	11.486	12.434	12.824	12.937
8.074	8.040	8.987	9.147	8.954
5.892	5.849	6.788	6.898	7.048
4.847	4.820	5.636	5.644	6.662
4.345	4.317	5.080	5.075	6.056
10.763	10.719	11.593	11.894	11.978
11.197	11.161	12.108	12.531	12.669
9.633	9.581	10.373	10.610	10.99
11.019	10.984	11.929	12.339	12.493
9.210	9.160	9.952	10.220	10.389
10.821	10.793	11.729	12.162	12.398
10.822	10.793	11.728	12.147	12.496
8.385	8.359	9.360	9.577	9.313
8.290	8.255	9.191	9.370	9.208
7.434	7.397	8.309	8.410	8.433
7.907	7.873	8.820	8.982	8.709
7.452	7.410	8.292	8.398	8.207
7.745	7.713	8.661	8.813	8.775
7.723	7.691	8.635	8.786	8.739
6.146	6.106	7.007	7.167	7.379
5.992	5.956	6.887	7.042	7.227
5.970	5.926	6.794	6.875	6.969
5.876	5.836	6.765	6.873	7.004
5.983	5.933	6.796	6.844	6.761

*Diffusion Monte Carlo (DMC) data from reference *Chem*

5.804	5.764	6.688	6.798	6.896
5.150	5.124	5.885	5.977	6.958
4.999	4.977	5.761	5.851	6.839
5.043	5.018	5.833	5.870	6.699
4.951	4.924	5.734	5.779	6.703
4.826	4.796	5.597	5.568	6.673
4.803	4.777	5.576	5.610	6.642
4.540	4.536	5.222	5.393	6.324
4.441	4.436	5.170	5.309	6.225
4.401	4.364	5.193	5.194	6.007
4.489	4.460	5.226	5.232	5.983
4.224	4.187	5.022	5.001	6.042
4.293	4.279	5.009	5.118	6.111

DOUBLE HYBRID GGA

B2PLYP	MPW2PLYP	PBEQIDH	PBE0DH
1.415	1.451	0.503	0.113
0.880	0.914	0.033	0.193
1.156	1.199	0.260	0.150
1.815	1.842	1.026	1.018
1.711	1.739	0.976	0.981
1.215	1.259	0.385	0.084
1.472	1.508	0.561	0.138
1.357	1.409	0.617	0.380
1.474	1.509	0.564	0.154

1.179	1.229	0.437	0.169
1.577	1.605	0.669	0.236
1.674	1.703	0.768	0.349
0.928	0.954	0.047	0.264
0.918	0.953	0.017	0.162
0.999	1.036	0.124	0.023
0.802	0.836	0.111	0.273
0.755	0.797	0.085	0.191
1.030	1.062	0.114	0.038
1.016	1.048	0.104	0.047
1.233	1.273	0.372	0.212
1.235	1.271	0.340	0.185
0.999	1.043	0.175	0.094
1.128	1.168	0.239	0.131
0.778	0.828	0.035	0.083
1.092	1.132	0.208	0.098
1.808	1.834	1.073	0.981
1.840	1.862	1.078	0.988
1.656	1.681	0.866	0.829
1.752	1.779	0.969	0.924
1.847	1.877	1.076	1.105
1.839	1.865	1.066	1.032
1.784	1.788	1.102	0.931
1.784	1.789	1.055	0.916
1.606	1.643	0.814	0.813
1.494	1.523	0.757	0.751
1.818	1.855	1.020	1.041
1.818	1.832	1.102	0.993

DOUBLE HYBRID GGA			
B2PLYP	MPW2PLYP	PBEQIDH	PBE0DH
1.375	1.408	0.561	0.461

DOUBLE HYBRID GGA			
B2PLYP	MPW2PLYP	PBEQIDH	PBE0DH
-1.415	-1.451	-0.503	-0.113
-0.880	-0.914	0.033	0.193
-1.156	-1.199	-0.260	-0.150
-1.815	-1.842	-1.026	-1.018
-1.711	-1.739	-0.976	-0.981
-1.215	-1.259	-0.385	-0.084
-1.472	-1.508	-0.561	-0.138
-1.357	-1.409	-0.617	-0.380
-1.474	-1.509	-0.564	-0.154
-1.179	-1.229	-0.437	-0.169
-1.577	-1.605	-0.669	-0.236
-1.674	-1.703	-0.768	-0.349
-0.928	-0.954	0.047	0.264
-0.918	-0.953	-0.017	0.162
-0.999	-1.036	-0.124	-0.023

	-0.802	-0.836	0.111	0.273
	-0.755	-0.797	0.085	0.191
	-1.030	-1.062	-0.114	0.038
	-1.016	-1.048	-0.104	0.047
	-1.233	-1.273	-0.372	-0.212
	-1.235	-1.271	-0.340	-0.185
	-0.999	-1.043	-0.175	-0.094
	-1.128	-1.168	-0.239	-0.131
	-0.778	-0.828	0.035	0.083
	-1.092	-1.132	-0.208	-0.098
	-1.808	-1.834	-1.073	-0.981
	-1.840	-1.862	-1.078	-0.988
	-1.656	-1.681	-0.866	-0.829
	-1.752	-1.779	-0.969	-0.924
	-1.847	-1.877	-1.076	-1.105
	-1.839	-1.865	-1.066	-1.032
	-1.784	-1.788	-1.102	-0.931
	-1.784	-1.789	-1.055	-0.916
	-1.606	-1.643	-0.814	-0.813
	-1.494	-1.523	-0.757	-0.751
	-1.818	-1.855	-1.020	-1.041
	-1.818	-1.832	-1.102	-0.993

**DOUBLE HYBRID GGA**				
**B2PLYP**	**MPW2PLYP**	**PBEQIDH**	**PBE0DH**	
	-1.375	-1.408	-0.544	-0.394

. *Phys.* **2023**, *565* , 111767.

Table S3. Empirical dispersion correction parameters by Grimme for D3 (with and without Becke-Johnson (B

					D3		
	GGA			META-GGA	HYBRID GGA		
Parameters	PBE	BP86	BPBE	TPSS	B3LYP	PBE0	B3PW91
S6	1.0000	1.0000	1.0000	1.0000	1.0000	1.0000	1.0000
SR6	1.2170	1.1390	1.0870	1.1660	1.2610	1.2870	1.1760
S8	0.7220	1.6830	2.0330	1.1050	1.7030	0.9280	1.7750

					D3-BJ		
	GGA			META-GGA	HYBRID GGA		
Parameters	PBE	BP86	BPBE	TPSS	B3LYP	PBE0	B3PW91
S6	1.0000	1.0000	1.0000	1.0000	1.0000	1.0000	1.0000
S8	0.7875	3.2822	4.0728	1.9435	1.9889	1.2177	2.8524
A1	0.4289	0.3946	0.4567	0.4535	0.3981	0.4145	0.4312
A2	4.4407	4.8516	4.3908	4.4752	4.4211	4.8593	4.4693

J) damping), available in the Gaussian 09/16 packages, for nin

RANGE-SEPARATED HYBRID GGA	DOUBLE HYBRID GGA
LC-ωPBE	B2PLYP
1.0000	0.6400
1.3550	1.4270
1.2790	1.0220

RANGE-SEPARATED HYBRID GGA	DOUBLE HYBRID GGA
LC-ωPBE	B2PLYP
1.0000	0.6400
1.3547	0.9147
0.4421	0.3065
4.6788	5.0570

Table S4. Atomization energies calculated with different exchange-correlation functionals plus D3-BJ emp

	Density Functional			
	GGA			META-GGA
Systems	PBE	BP86	BPBE	TPSS
Li_{13}	13.039	13.869	13.736	14.126
Na_{13}	9.246	10.298	10.111	10.432
K_{13}	6.811	7.763	7.559	8.094
Rb_{13}	6.117	7.202	6.837	7.369
Cs_{13}	5.442	6.436	6.018	6.634
$NaLi_{12}$(c)	11.939	12.797	12.616	13.028
$NaLi_{12}$(f)	12.727	13.573	13.440	13.823
KLi_{12}(c)	10.624	11.542	11.284	11.733
KLi_{12}(f)	12.524	13.375	13.233	13.625
$RbLi_{12}$(c)	9.898	10.789	10.489	11.012
$RbLi_{12}$(f)	12.457	13.314	13.162	13.556
$CsLi_{12}$(f)	12.444	13.302	13.141	13.535
$LiNa_{12}$(c)	9.597	10.593	10.452	10.793
$LiNa_{12}$(f)	9.461	10.502	10.316	10.638
KNa_{12}(c)	8.467	9.517	9.280	9.658
KNa_{12}(f)	9.053	10.096	9.908	10.248
$RbNa_{12}$(c)	8.158	9.208	8.942	9.347
$RbNa_{12}$(f)	8.995	10.040	9.843	10.186
$CsNa_{12}$(f)	8.964	10.009	9.804	10.150
LiK_{12}(f)	7.114	8.087	7.864	8.389
NaK_{12}(f)	6.961	7.926	7.715	8.239
RbK_{12}(c)	6.690	7.665	7.421	7.966
RbK_{12}(f)	6.755	7.713	7.500	8.036
CsK_{12}(c)	6.575	7.598	7.303	7.850
CsK_{12}(f)	6.712	7.663	7.447	7.989
$LiRb_{12}$(f)	6.426	7.521	7.146	7.683
$NaRb_{12}$(f)	6.311	7.402	7.040	7.568
KRb_{12}(c)	6.235	7.308	6.979	7.496
KRb_{12}(f)	6.169	7.248	6.892	7.423
$CsRb_{12}$(c)	6.003	7.094	6.693	7.249
$CsRb_{12}$(f)	6.075	7.152	6.785	7.322
$LiCs_{12}$(f)	5.807	6.833	6.398	7.037
$NaCs_{12}$(f)	5.706	6.722	6.303	6.932
KCs_{12}(c)	5.582	6.541	6.204	6.786
KCs_{12}(f)	5.549	6.548	6.138	6.753
$RbCs_{12}$(c)	5.508	6.487	6.110	6.708
$RbCs_{12}$(f)	5.497	6.502	6.083	6.695

	Density Functional			
	GGA			META-GGA

	PBE	BP86	BPBE	TPSS
**SD**	2.424	2.370	2.449	2.371

Absolute deviations

	Density Functional			
	GGA			META-GGA
**Systems**	**PBE**	**BP86**	**BPBE**	**TPSS**
Li_{13}	0.102	0.932	0.799	1.189
Na_{13}	0.292	1.344	1.157	1.478
K_{13}	0.237	0.715	0.511	1.046
Rb_{13}	0.545	0.540	0.175	0.707
Cs_{13}	0.614	0.380	0.038	0.578
$NaLi_{12}$(c)	0.039	0.819	0.638	1.050
$NaLi_{12}$(f)	0.058	0.904	0.771	1.154
KLi_{12}(c)	0.366	0.552	0.294	0.743
KLi_{12}(f)	0.031	0.882	0.740	1.132
$RbLi_{12}$(c)	0.491	0.400	0.100	0.623
$RbLi_{12}$(f)	0.059	0.916	0.764	1.158
$CsLi_{12}$(f)	0.052	0.806	0.645	1.039
$LiNa_{12}$(c)	0.284	1.280	1.139	1.480
$LiNa_{12}$(f)	0.253	1.294	1.108	1.430
KNa_{12}(c)	0.034	1.084	0.847	1.225
KNa_{12}(f)	0.344	1.387	1.199	1.539
$RbNa_{12}$(c)	0.049	1.001	0.735	1.140
$RbNa_{12}$(f)	0.220	1.265	1.068	1.411
$CsNa_{12}$(f)	0.225	1.270	1.065	1.411
LiK_{12}(f)	0.265	0.708	0.485	1.010
NaK_{12}(f)	0.266	0.699	0.488	1.012
RbK_{12}(c)	0.279	0.696	0.452	0.997
RbK_{12}(f)	0.249	0.709	0.496	1.032
CsK_{12}(c)	0.186	0.837	0.542	1.089
CsK_{12}(f)	0.184	0.767	0.551	1.093
$LiRb_{12}$(f)	0.532	0.563	0.188	0.725
$NaRb_{12}$(f)	0.528	0.563	0.201	0.729
KRb_{12}(c)	0.464	0.609	0.280	0.797
KRb_{12}(f)	0.534	0.545	0.189	0.720
$CsRb_{12}$(c)	0.670	0.421	0.020	0.576
$CsRb_{12}$(f)	0.567	0.510	0.143	0.680
$LiCs_{12}$(f)	0.517	0.509	0.074	0.713
$NaCs_{12}$(f)	0.519	0.497	0.078	0.707
KCs_{12}(c)	0.425	0.534	0.197	0.779
KCs_{12}(f)	0.434	0.565	0.155	0.770
$RbCs_{12}$(c)	0.534	0.445	0.068	0.666
$RbCs_{12}$(f)	0.614	0.391	0.028	0.584

	Density Functional			
	GGA			META-GGA
	PBE	BP86	BPBE	TPSS
**MAD**	0.326	0.766	0.498	0.979
**MAE**	0.670	1.387	1.199	1.539
**SD(MAD)**	0.194	0.295	0.369	0.286

Signed deviations

	Density Functional			
	GGA			META-GGA
Systems	PBE	BP86	BPBE	TPSS
Li_{13}	0.102	0.932	0.799	1.189
Na_{13}	0.292	1.344	1.157	1.478
K_{13}	-0.237	0.715	0.511	1.046
Rb_{13}	-0.545	0.540	0.175	0.707
Cs_{13}	-0.614	0.380	-0.038	0.578
$NaLi_{12}$(c)	-0.039	0.819	0.638	1.050
$NaLi_{12}$(f)	0.058	0.904	0.771	1.154
KLi_{12}(c)	-0.366	0.552	0.294	0.743
KLi_{12}(f)	0.031	0.882	0.740	1.132
$RbLi_{12}$(c)	-0.491	0.400	0.100	0.623
$RbLi_{12}$(f)	0.059	0.916	0.764	1.158
$CsLi_{12}$(f)	-0.052	0.806	0.645	1.039
$LiNa_{12}$(c)	0.284	1.280	1.139	1.480
$LiNa_{12}$(f)	0.253	1.294	1.108	1.430
KNa_{12}(c)	0.034	1.084	0.847	1.225
KNa_{12}(f)	0.344	1.387	1.199	1.539
$RbNa_{12}$(c)	-0.049	1.001	0.735	1.140
$RbNa_{12}$(f)	0.220	1.265	1.068	1.411
$CsNa_{12}$(f)	0.225	1.270	1.065	1.411
LiK_{12}(f)	-0.265	0.708	0.485	1.010
NaK_{12}(f)	-0.266	0.699	0.488	1.012
RbK_{12}(c)	-0.279	0.696	0.452	0.997
RbK_{12}(f)	-0.249	0.709	0.496	1.032
CsK_{12}(c)	-0.186	0.837	0.542	1.089
CsK_{12}(f)	-0.184	0.767	0.551	1.093
$LiRb_{12}$(f)	-0.532	0.563	0.188	0.725
$NaRb_{12}$(f)	-0.528	0.563	0.201	0.729
KRb_{12}(c)	-0.464	0.609	0.280	0.797
KRb_{12}(f)	-0.534	0.545	0.189	0.720
$CsRb_{12}$(c)	-0.670	0.421	0.020	0.576
$CsRb_{12}$(f)	-0.567	0.510	0.143	0.680

$LiCs_{12}$(f)	-0.517	0.509	0.074	0.713
$NaCs_{12}$(f)	-0.519	0.497	0.078	0.707
KCs_{12}(c)	-0.425	0.534	0.197	0.779
KCs_{12}(f)	-0.434	0.565	0.155	0.770
$RbCs_{12}$(c)	-0.534	0.445	0.068	0.666
$RbCs_{12}$(f)	-0.614	0.391	-0.028	0.584

	**Density Functional**			
	**GGA**			**META-GGA**
	**PBE**	**BP86**	**BPBE**	**TPSS**
**MSD**	-0.223	0.766	0.494	0.979
**SD(MSD)**	0.307	0.295	0.374	0.286

ɔirical dispersion corrections and the def2-TZVP basis set along with Mean Absı

HYBRID GGA			
**B3LYP**		**PBE0**	**B3PW91**
	12.170	12.859	13.441
	8.607	9.126	9.801
	6.239	6.824	7.362
	5.720	6.146	6.705
	5.063	5.483	5.950
	11.146	11.760	12.339
	11.872	12.552	13.141
	9.986	10.461	11.054
	11.685	12.355	12.940
	9.251	9.750	10.291
	11.626	12.290	12.873
	11.618	12.274	12.853
	8.860	9.481	10.131
	8.816	9.334	10.005
	7.905	8.380	9.026
	8.413	8.945	9.607
	7.613	8.079	8.709
	8.359	8.888	9.546
	8.333	8.857	9.510
	6.568	7.106	7.661
	6.399	6.967	7.515
	6.167	6.712	7.250
	6.190	6.770	7.307
	6.128	6.599	7.155
	6.147	6.728	7.258
	6.048	6.437	7.006
	5.918	6.335	6.903
	5.795	6.262	6.825
	5.764	6.197	6.757
	5.654	6.038	6.590
	5.675	6.104	6.656
	5.474	5.835	6.320
	5.349	5.748	6.229
	5.029	5.538	6.009
	5.169	5.590	6.064
	4.940	5.428	5.895
	5.123	5.538	6.012

HYBRID GGA

B3LYP		PBE0	B3PW91
	2.277	2.348	2.383

HYBRID GGA

B3LYP		PBE0	B3PW91
	0.767	0.078	0.504
	0.347	0.172	0.847
	0.809	0.224	0.314
	0.942	0.516	0.043
	0.993	0.573	0.106
	0.832	0.218	0.361
	0.797	0.117	0.472
	1.004	0.529	0.064
	0.808	0.138	0.447
	1.138	0.639	0.098
	0.772	0.108	0.475
	0.878	0.222	0.357
	0.453	0.168	0.818
	0.392	0.126	0.797
	0.528	0.053	0.593
	0.296	0.236	0.898
	0.594	0.128	0.502
	0.416	0.113	0.771
	0.406	0.118	0.771
	0.811	0.273	0.282
	0.828	0.260	0.288
	0.802	0.257	0.281
	0.814	0.234	0.303
	0.633	0.162	0.394
	0.749	0.168	0.362
	0.910	0.521	0.048
	0.921	0.504	0.064
	0.904	0.437	0.126
	0.939	0.506	0.054
	1.019	0.635	0.083
	0.967	0.538	0.014
	0.850	0.489	0.004
	0.876	0.477	0.004
	0.978	0.469	0.002
	0.814	0.393	0.081
	1.102	0.614	0.147
	0.988	0.573	0.099

HYBRID GGA			
B3LYP	PBE0	B3PW91	
	0.786	0.324	0.321
	1.138	0.639	0.898
	0.215	0.188	0.274

HYBRID GGA			
B3LYP	PBE0	B3PW91	
	-0.767	-0.078	0.504
	-0.347	0.172	0.847
	-0.809	-0.224	0.314
	-0.942	-0.516	0.043
	-0.993	-0.573	-0.106
	-0.832	-0.218	0.361
	-0.797	-0.117	0.472
	-1.004	-0.529	0.064
	-0.808	-0.138	0.447
	-1.138	-0.639	-0.098
	-0.772	-0.108	0.475
	-0.878	-0.222	0.357
	-0.453	0.168	0.818
	-0.392	0.126	0.797
	-0.528	-0.053	0.593
	-0.296	0.236	0.898
	-0.594	-0.128	0.502
	-0.416	0.113	0.771
	-0.406	0.118	0.771
	-0.811	-0.273	0.282
	-0.828	-0.260	0.288
	-0.802	-0.257	0.281
	-0.814	-0.234	0.303
	-0.633	-0.162	0.394
	-0.749	-0.168	0.362
	-0.910	-0.521	0.048
	-0.921	-0.504	0.064
	-0.904	-0.437	0.126
	-0.939	-0.506	0.054
	-1.019	-0.635	-0.083
	-0.967	-0.538	0.014

-0.850	-0.489	-0.004
-0.876	-0.477	0.004
-0.978	-0.469	0.002
-0.814	-0.393	0.081
-1.102	-0.614	-0.147
-0.988	-0.573	-0.099

HYBRID GGA

**B3LYP**	**PBE0**	**B3PW91**
-0.786	-0.274	0.292
0.215	0.256	0.305

olute Deviations (MADs), Maximum Absolute Errors (MAEs), and Mean Signed Deviations (MSDs) compared to

RANGE-SEPARATED HYBRID GGA	DOUBLE HYBRID GGA	DMC*
**LC-ωPBE**	**B2PLYP**	**REFERENCE**
12.469	11.807	12.937
8.848	8.680	8.954
6.795	6.467	7.048
6.183	6.036	6.662
5.556	5.408	6.056
11.338	10.924	11.978
12.216	11.531	12.669
10.070	9.865	10.99
12.025	11.348	12.493
9.373	9.200	10.389
11.966	11.295	12.398
11.942	11.282	12.496
9.227	8.879	9.313
9.045	8.867	9.208
8.157	8.064	8.433
8.693	8.495	8.709
7.873	7.809	8.207
8.639	8.445	8.775
8.612	8.420	8.739
7.063	6.705	7.379
6.955	6.566	7.227
6.713	6.438	6.969
6.755	6.420	7.004
6.612	6.426	6.761
6.722	6.364	6.896
6.499	6.199	6.958
6.425	6.103	6.839
6.292	6.061	6.699
6.242	6.066	6.703
6.089	6.008	6.673
6.157	5.972	6.642
5.997	5.556	6.324
5.932	5.474	6.225
5.540	5.343	6.007
5.643	5.360	5.983
5.415	5.294	6.042
5.601	5.345	6.111

*Diffusion Monte Carlo

RANGE-SEPARATED HYBRID GGA	DOUBLE HYBRID GGA

LC-ωPBE	B2PLYP
2.182	2.075

RANGE-SEPARATED HYBRID GGA	DOUBLE HYBRID GGA
LC-ωPBE	B2PLYP
0.468	1.130
0.106	0.274
0.253	0.581
0.479	0.626
0.500	0.648
0.640	1.054
0.453	1.138
0.920	1.125
0.468	1.145
1.016	1.189
0.432	1.103
0.554	1.214
0.086	0.434
0.163	0.341
0.276	0.369
0.016	0.214
0.334	0.398
0.136	0.330
0.127	0.319
0.316	0.674
0.272	0.661
0.256	0.531
0.249	0.584
0.149	0.335
0.174	0.532
0.459	0.759
0.414	0.736
0.407	0.638
0.461	0.637
0.584	0.665
0.485	0.670
0.327	0.768
0.293	0.751
0.467	0.664
0.340	0.623
0.627	0.748
0.510	0.766

RANGE-SEPARATED HYBRID GGA	DOUBLE HYBRID GGA
LC-ωPBE	B2PLYP
0.384	0.686
1.016	1.214
0.211	0.280

RANGE-SEPARATED HYBRID GGA	DOUBLE HYBRID GGA
LC-ωPBE	B2PLYP
-0.468	-1.130
-0.106	-0.274
-0.253	-0.581
-0.479	-0.626
-0.500	-0.648
-0.640	-1.054
-0.453	-1.138
-0.920	-1.125
-0.468	-1.145
-1.016	-1.189
-0.432	-1.103
-0.554	-1.214
-0.086	-0.434
-0.163	-0.341
-0.276	-0.369
-0.016	-0.214
-0.334	-0.398
-0.136	-0.330
-0.127	-0.319
-0.316	-0.674
-0.272	-0.661
-0.256	-0.531
-0.249	-0.584
-0.149	-0.335
-0.174	-0.532
-0.459	-0.759
-0.414	-0.736
-0.407	-0.638
-0.461	-0.637
-0.584	-0.665
-0.485	-0.670

-0.327	-0.768
-0.293	-0.751
-0.467	-0.664
-0.340	-0.623
-0.627	-0.748
-0.510	-0.766

RANGE-SEPARATED HYBRID GGA LC-ωPBE	DOUBLE HYBRID GGA B2PLYP
-0.384	-0.686
0.211	0.280

reference data (in eV) .

) (DMC) data from reference *Chem. Phys.* **2023**, *565* , 111767.

Table S5. Atomization energies calculated with different exchange-correlation functionals (with and w

	**Without Empirical Dispersion Corrections**						
	**Density Functional**						
	**GGA**			**META-GGA**	**HYBRID GGA**		
**Systems**	**PBE**	**BP86**	**BPBE**	**TPSS**	**B3LYP**	**PBE0**	**B3PW91**
Li_{13}	12.401	12.403	12.092	13.220	10.813	12.202	12.061
Na_{13}	8.568	8.478	8.253	9.432	7.036	8.373	8.216
K_{13}	6.276	6.187	5.999	7.263	4.994	6.186	6.043
Rb_{13}	5.605	5.556	5.339	6.579	4.429	5.516	5.401
Cs_{13}	5.014	4.980	4.750	5.968	3.953	4.940	4.834
$NaLi_{12}$(c)	11.325	11.350	11.021	12.152	9.823	11.120	10.996
$NaLi_{12}$(f)	12.085	12.078	11.776	12.908	10.497	11.887	11.743
KLi_{12}(c)	10.029	10.106	9.725	10.880	8.690	9.834	9.738
KLi_{12}(f)	11.889	11.888	11.583	12.719	10.326	11.695	11.555
$RbLi_{12}$(c)	9.351	9.438	9.040	10.221	8.052	9.164	9.068
$RbLi_{12}$(f)	11.827	11.827	11.521	12.655	10.271	11.633	11.493
$CsLi_{12}$(f)	11.820	11.823	11.511	12.641	10.274	11.621	11.483
$LiNa_{12}$(c)	8.919	8.804	8.600	9.794	7.307	8.733	8.557
$LiNa_{12}$(f)	8.787	8.709	8.474	9.646	7.261	8.588	8.436
KNa_{12}(c)	7.829	7.771	7.514	8.710	6.417	7.663	7.518
KNa_{12}(f)	8.388	8.296	8.075	9.263	6.869	8.202	8.044
$RbNa_{12}$(c)	7.542	7.501	7.234	8.431	6.169	7.381	7.248
$RbNa_{12}$(f)	8.335	8.244	8.023	9.208	6.821	8.149	7.992
$CsNa_{12}$(f)	8.310	8.223	7.997	9.180	6.807	8.123	7.968
LiK_{12}(f)	6.571	6.511	6.295	7.552	5.309	6.465	6.334
NaK_{12}(f)	6.417	6.334	6.138	7.399	5.133	6.322	6.181
RbK_{12}(c)	6.163	6.094	5.886	7.150	4.927	6.081	5.947
RbK_{12}(f)	6.222	6.135	5.946	7.209	4.945	6.134	5.992
CsK_{12}(c)	6.051	6.017	5.778	7.039	4.880	5.969	5.855
CsK_{12}(f)	6.186	6.099	5.910	7.171	4.915	6.098	5.956
$LiRb_{12}$(f)	5.909	5.890	5.642	6.888	4.759	5.807	5.701
$NaRb_{12}$(f)	5.791	5.749	5.524	6.767	4.614	5.699	5.585
KRb_{12}(c)	5.711	5.643	5.446	6.686	4.488	5.620	5.495
KRb_{12}(f)	5.654	5.604	5.388	6.630	4.474	5.566	5.449
$CsRb_{12}$(c)	5.507	5.484	5.240	6.483	4.397	5.424	5.322
$CsRb_{12}$(f)	5.569	5.520	5.304	6.541	4.398	5.481	5.365
$LiCs_{12}$(f)	5.370	5.377	5.108	6.358	4.354	5.285	5.191
$NaCs_{12}$(f)	5.266	5.247	5.003	6.248	4.217	5.193	5.089
KCs_{12}(c)	5.137	5.058	4.882	6.090	3.896	4.977	4.856
KCs_{12}(f)	5.114	5.082	4.849	6.075	4.048	5.039	4.933

$RbCs_{12}$(c)	5.071	5.009	4.811	6.026	3.812	4.874	4.757
$RbCs_{12}$(f)	5.064	5.032	4.799	6.021	4.001	4.988	4.883

vithout D3-BJ empirical dispersion corrections) and the def2-TZVP basis set along with the effect

RANGE-SEPARATED HYBRID GGA	DOUBLE HYBRID GGA	
LC-ωPBE	B2PLYP	Systems
11.639	10.871	Li_{13}
7.829	7.500	Na_{13}
5.897	5.552	K_{13}
5.255	4.980	Rb_{13}
4.733	4.470	Cs_{13}
10.520	9.997	$NaLi_{12}$(c)
11.370	10.577	$NaLi_{12}$(f)
9.260	8.941	KLi_{12}(c)
11.182	10.405	KLi_{12}(f)
8.609	8.332	$RbLi_{12}$(c)
11.124	10.351	$RbLi_{12}$(f)
11.105	10.344	$CsLi_{12}$(f)
8.221	7.728	$LiNa_{12}$(c)
8.039	7.704	$LiNa_{12}$(f)
7.178	6.941	KNa_{12}(c)
7.683	7.336	KNa_{12}(f)
6.915	6.701	$RbNa_{12}$(c)
7.632	7.285	$RbNa_{12}$(f)
7.610	7.266	$CsNa_{12}$(f)
6.165	5.779	LiK_{12}(f)
6.049	5.632	NaK_{12}(f)
5.818	5.508	RbK_{12}(c)
5.856	5.499	RbK_{12}(f)
5.713	5.469	CsK_{12}(c)
5.830	5.453	CsK_{12}(f)
5.580	5.154	$LiRb_{12}$(f)
5.494	5.043	$NaRb_{12}$(f)
5.354	5.010	KRb_{12}(c)
5.316	5.019	KRb_{12}(f)
5.182	4.968	$CsRb_{12}$(c)
5.237	4.925	$CsRb_{12}$(f)
5.174	4.617	$LiCs_{12}$(f)
5.098	4.522	$NaCs_{12}$(f)
4.700	4.411	KCs_{12}(c)
4.815	4.422	KCs_{12}(f)

	4.581	4.351	$RbCs_{12}$(c)
	4.771	4.397	$RbCs_{12}$(f)

of these corrections (in eV) .

With D3-BJ Empirical Dispersion Corrections						
Density Functional						
**GGA**			**META-GGA**	**HYBRID GGA**		
**PBE**	**BP86**	**BPBE**	**TPSS**	**B3LYP**	**PBE0**	**B3PW91**
13.039	13.869	13.736	14.126	12.170	12.859	13.441
9.246	10.298	10.111	10.432	8.607	9.126	9.801
6.811	7.763	7.559	8.094	6.239	6.824	7.362
6.117	7.202	6.837	7.369	5.720	6.146	6.705
5.442	6.436	6.018	6.634	5.063	5.483	5.950
11.939	12.797	12.616	13.028	11.146	11.760	12.339
12.727	13.573	13.440	13.823	11.872	12.552	13.141
10.624	11.542	11.284	11.733	9.986	10.461	11.054
12.524	13.375	13.233	13.625	11.685	12.355	12.940
9.898	10.789	10.489	11.012	9.251	9.750	10.291
12.457	13.314	13.162	13.556	11.626	12.290	12.873
12.444	13.302	13.141	13.535	11.618	12.274	12.853
9.597	10.593	10.452	10.793	8.860	9.481	10.131
9.461	10.502	10.316	10.638	8.816	9.334	10.005
8.467	9.517	9.280	9.658	7.905	8.380	9.026
9.053	10.096	9.908	10.248	8.413	8.945	9.607
8.158	9.208	8.942	9.347	7.613	8.079	8.709
8.995	10.040	9.843	10.186	8.359	8.888	9.546
8.964	10.009	9.804	10.150	8.333	8.857	9.510
7.114	8.087	7.864	8.389	6.568	7.106	7.661
6.961	7.926	7.715	8.239	6.399	6.967	7.515
6.690	7.665	7.421	7.966	6.167	6.712	7.250
6.755	7.713	7.500	8.036	6.190	6.770	7.307
6.575	7.598	7.303	7.850	6.128	6.599	7.155
6.712	7.663	7.447	7.989	6.147	6.728	7.258
6.426	7.521	7.146	7.683	6.048	6.437	7.006
6.311	7.402	7.040	7.568	5.918	6.335	6.903
6.235	7.308	6.979	7.496	5.795	6.262	6.825
6.169	7.248	6.892	7.423	5.764	6.197	6.757
6.003	7.094	6.693	7.249	5.654	6.038	6.590
6.075	7.152	6.785	7.322	5.675	6.104	6.656
5.807	6.833	6.398	7.037	5.474	5.835	6.320
5.706	6.722	6.303	6.932	5.349	5.748	6.229
5.582	6.541	6.204	6.786	5.029	5.538	6.009
5.549	6.548	6.138	6.753	5.169	5.590	6.064

	5.508	6.487		6.110	6.708		4.940	5.428	5.895
	5.497	6.502		6.083	6.695		5.123	5.538	6.012

RANGE-SEPARATED HYBRID GGA	DOUBLE HYBRID GGA		Effect of D3-BJ dispersion c[illegible]<br>Density Functional<br>GGA	
LC-ωPBE	B2PLYP	Systems	PBE	BP86
12.469	11.807	Li_{13}	0.638	1.467
8.848	8.680	Na_{13}	0.677	1.820
6.795	6.467	K_{13}	0.535	1.576
6.183	6.036	Rb_{13}	0.512	1.647
5.556	5.408	Cs_{13}	0.427	1.456
11.338	10.924	$NaLi_{12}$(c)	0.614	1.447
12.216	11.531	$NaLi_{12}$(f)	0.642	1.495
10.070	9.865	KLi_{12}(c)	0.595	1.436
12.025	11.348	KLi_{12}(f)	0.634	1.487
9.373	9.200	$RbLi_{12}$(c)	0.547	1.351
11.966	11.295	$RbLi_{12}$(f)	0.630	1.486
11.942	11.282	$CsLi_{12}$(f)	0.625	1.478
9.227	8.879	$LiNa_{12}$(c)	0.678	1.790
9.045	8.867	$LiNa_{12}$(f)	0.674	1.794
8.157	8.064	KNa_{12}(c)	0.639	1.746
8.693	8.495	KNa_{12}(f)	0.665	1.800
7.873	7.809	$RbNa_{12}$(c)	0.616	1.707
8.639	8.445	$RbNa_{12}$(f)	0.660	1.797
8.612	8.420	$CsNa_{12}$(f)	0.654	1.786
7.063	6.705	LiK_{12}(f)	0.543	1.577
6.955	6.566	NaK_{12}(f)	0.544	1.592
6.713	6.438	RbK_{12}(c)	0.527	1.572
6.755	6.420	RbK_{12}(f)	0.532	1.578
6.612	6.426	CsK_{12}(c)	0.524	1.582
6.722	6.364	CsK_{12}(f)	0.526	1.564
6.499	6.199	$LiRb_{12}$(f)	0.517	1.631
6.425	6.103	$NaRb_{12}$(f)	0.520	1.653
6.292	6.061	KRb_{12}(c)	0.524	1.664
6.242	6.066	KRb_{12}(f)	0.514	1.644
6.089	6.008	$CsRb_{12}$(c)	0.496	1.610
6.157	5.972	$CsRb_{12}$(f)	0.506	1.633
5.997	5.556	$LiCs_{12}$(f)	0.438	1.456
5.932	5.474	$NaCs_{12}$(f)	0.440	1.475
5.540	5.343	KCs_{12}(c)	0.445	1.483
5.643	5.360	KCs_{12}(f)	0.435	1.466

	5.415	5.294	$RbCs_{12}$(c)		0.438	1.477
	5.601	5.345	$RbCs_{12}$(f)		0.433	1.470

	**PBE**		**BP86**
**Minimun**		0.427	1.351
**Maximun**		0.678	1.820

orrections

	META-GGA	HYBRID GGA			RANGE-SEPARATED HYBRID GGA
BPBE	TPSS	B3LYP	PBE0	B3PW91	LC-ωPBE
1.644	0.906	1.357	0.657	1.380	0.831
1.858	0.999	1.571	0.753	1.585	1.020
1.560	0.831	1.245	0.638	1.318	0.899
1.498	0.790	1.291	0.630	1.304	0.927
1.268	0.666	1.110	0.544	1.116	0.823
1.596	0.876	1.323	0.639	1.343	0.818
1.664	0.915	1.375	0.666	1.398	0.846
1.559	0.854	1.296	0.627	1.316	0.810
1.650	0.907	1.359	0.661	1.386	0.843
1.448	0.792	1.199	0.586	1.223	0.764
1.642	0.901	1.354	0.658	1.380	0.841
1.630	0.895	1.344	0.653	1.370	0.837
1.852	0.999	1.553	0.748	1.574	1.005
1.842	0.992	1.555	0.746	1.570	1.006
1.766	0.948	1.488	0.718	1.508	0.980
1.833	0.985	1.544	0.743	1.563	1.010
1.707	0.916	1.445	0.697	1.461	0.958
1.821	0.978	1.538	0.739	1.554	1.007
1.806	0.970	1.526	0.734	1.543	1.002
1.569	0.837	1.259	0.641	1.327	0.898
1.577	0.841	1.266	0.645	1.334	0.906
1.535	0.816	1.240	0.631	1.303	0.895
1.553	0.827	1.245	0.636	1.315	0.899
1.525	0.811	1.248	0.630	1.301	0.899
1.537	0.818	1.232	0.630	1.301	0.892
1.504	0.795	1.290	0.630	1.305	0.919
1.516	0.801	1.304	0.636	1.318	0.931
1.533	0.809	1.308	0.642	1.329	0.938
1.504	0.794	1.290	0.631	1.307	0.926
1.453	0.766	1.258	0.614	1.268	0.907
1.481	0.781	1.278	0.624	1.291	0.920
1.290	0.679	1.120	0.550	1.130	0.823
1.300	0.684	1.133	0.555	1.140	0.833
1.322	0.695	1.133	0.561	1.153	0.840
1.289	0.678	1.120	0.551	1.131	0.829

1.298	0.682	1.128	0.554	1.139	0.835
1.284	0.674	1.123	0.550	1.129	0.830

BPBE	TPSS	B3LYP	PBE0	B3PW91	LC-ωPBE
1.268	0.666	1.110	0.544	1.116	0.764
1.858	0.999	1.571	0.753	1.585	1.020

DOUBLE HYBRID GGA
B2PLYP
0.936
1.181
0.915
1.056
0.938
0.927
0.954
0.924
0.943
0.869
0.944
0.938
1.151
1.163
1.123
1.159
1.109
1.160
1.154
0.926
0.934
0.930
0.921
0.957
0.911
1.045
1.061
1.051
1.047
1.040
1.046
0.940
0.952
0.932
0.938

0.943			
0.948			

B2PLYP

0.869	**Minimun**	0.427
1.181	**Maximun**	1.858

Table S6. Atomization energies calculated with different exchange-correlation functionals (without empirical disper

	def2-SVP								
	Density Functional								
	GGA			META-GGA	HYBRID GGA			RANGE-SE	DOUBLE H
**Systems**	**PBE**	**BP86**	**BPBE**	**TPSS**	**B3LYP**	**PBE0**	**B3PW91**	**LC-ωPBE**	**B2PLYP**
Li_{13}	13.408	13.260	13.122	14.183	11.604	13.028	12.903	12.134	11.522
Na_{13}	9.431	9.257	9.134	10.291	7.833	9.162	9.017	8.469	8.074
K_{13}	7.008	6.817	6.757	7.962	5.555	6.836	6.696	6.369	5.892
Rb_{13}	5.553	5.509	5.279	6.514	4.395	5.470	5.352	5.218	4.847
Cs_{13}	4.962	4.914	4.696	5.903	3.879	4.886	4.775	4.674	4.345
$NaLi_{12}$(c)	12.416	12.297	12.120	13.171	10.728	12.037	11.924	11.120	10.763
$NaLi_{12}$(f)	13.095	12.945	12.808	13.881	11.307	12.726	12.598	11.908	11.197
KLi_{12}(c)	11.061	11.006	10.771	11.835	9.549	10.701	10.622	9.840	9.633
KLi_{12}(f)	12.901	12.755	12.617	13.689	11.126	12.532	12.407	11.704	11.019
$RbLi_{12}$(c)	10.672	10.625	10.388	11.485	9.172	10.330	10.249	9.500	9.210
$RbLi_{12}$(f)	12.703	12.565	12.416	13.493	10.954	12.349	12.222	11.585	10.821
$CsLi_{12}$(f)	12.689	12.552	12.402	13.476	10.943	12.332	12.205	11.549	10.822
$LiNa_{12}$(c)	9.893	9.694	9.600	10.780	8.219	9.628	9.470	8.956	8.385
$LiNa_{12}$(f)	9.676	9.511	9.381	10.528	8.075	9.397	9.256	8.693	8.290
KNa_{12}(c)	8.597	8.452	8.302	9.478	7.122	8.371	8.235	7.772	7.434
KNa_{12}(f)	9.257	9.080	8.964	10.123	7.663	8.993	8.848	8.315	7.907
$RbNa_{12}$(c)	8.604	8.492	8.330	9.503	7.173	8.368	8.261	7.754	7.452
$RbNa_{12}$(f)	9.065	8.891	8.767	9.929	7.487	8.813	8.664	8.162	7.745
$CsNa_{12}$(f)	9.038	8.864	8.741	9.901	7.463	8.785	8.637	8.134	7.723
LiK_{12}(f)	7.317	7.153	7.065	8.259	5.882	7.124	6.995	6.632	6.146
NaK_{12}(f)	7.167	6.987	6.915	8.120	5.725	6.990	6.854	6.539	5.992
RbK_{12}(c)	6.987	6.835	6.734	7.944	5.613	6.799	6.677	6.293	5.970
RbK_{12}(f)	6.979	6.801	6.728	7.929	5.551	6.809	6.673	6.349	5.876
CsK_{12}(c)	6.937	6.817	6.692	7.899	5.625	6.754	6.653	6.263	5.983
CsK_{12}(f)	6.900	6.719	6.649	7.848	5.470	6.732	6.594	6.280	5.804
$LiRb_{12}$(f)	5.938	5.903	5.664	6.888	4.767	5.830	5.719	5.551	5.150
$NaRb_{12}$(f)	5.790	5.740	5.515	6.746	4.613	5.697	5.579	5.461	4.999
KRb_{12}(c)	5.816	5.766	5.546	6.758	4.642	5.723	5.605	5.446	5.043
KRb_{12}(f)	5.708	5.656	5.437	6.669	4.533	5.617	5.499	5.356	4.951
$CsRb_{12}$(c)	5.455	5.435	5.180	6.427	4.356	5.376	5.270	5.136	4.826
$CsRb_{12}$(f)	5.520	5.474	5.248	6.479	4.364	5.437	5.319	5.198	4.803
$LiCs_{12}$(f)	5.374	5.344	5.111	6.318	4.288	5.270	5.169	5.087	4.540
$NaCs_{12}$(f)	5.253	5.207	4.990	6.205	4.159	5.167	5.058	5.019	4.441
KCs_{12}(c)	5.243	5.158	4.986	6.160	3.951	5.035	4.921	4.712	4.401
KCs_{12}(f)	5.140	5.085	4.875	6.079	4.036	5.053	4.943	4.816	4.489

$RbCs_{12}$(c)	5.007	4.930	4.744	5.940	3.741	4.816	4.706	4.521	4.224
$RbCs_{12}$(f)	5.014	4.970	4.749	5.958	3.932	4.937	4.828	4.715	4.293

sion corrections) with the def2-SVP and def2-TZVP basis sets along with the effect of basis set increment (in

		def2-TZVP					
		Density Functional					
YBRID GGA		GGA			META-GGA	HYBRID GGA	
	Systems	PBE	BP86	BPBE	TPSS	B3LYP	PBE0
	Li_{13}	12.401	12.403	12.092	13.220	10.813	12.202
	Na_{13}	8.568	8.478	8.253	9.432	7.036	8.373
	K_{13}	6.276	6.187	5.999	7.263	4.994	6.186
	Rb_{13}	5.605	5.556	5.339	6.579	4.429	5.516
	Cs_{13}	5.014	4.980	4.750	5.968	3.953	4.940
	$NaLi_{12}$(c)	11.325	11.350	11.021	12.152	9.823	11.120
	$NaLi_{12}$(f)	12.085	12.078	11.776	12.908	10.497	11.887
	KLi_{12}(c)	10.029	10.106	9.725	10.880	8.690	9.834
	KLi_{12}(f)	11.889	11.888	11.583	12.719	10.326	11.695
	$RbLi_{12}$(c)	9.351	9.438	9.040	10.221	8.052	9.164
	$RbLi_{12}$(f)	11.827	11.827	11.521	12.655	10.271	11.633
	$CsLi_{12}$(f)	11.820	11.823	11.511	12.641	10.274	11.621
	$LiNa_{12}$(c)	8.919	8.804	8.600	9.794	7.307	8.733
	$LiNa_{12}$(f)	8.787	8.709	8.474	9.646	7.261	8.588
	KNa_{12}(c)	7.829	7.771	7.514	8.710	6.417	7.663
	KNa_{12}(f)	8.388	8.296	8.075	9.263	6.869	8.202
	$RbNa_{12}$(c)	7.542	7.501	7.234	8.431	6.169	7.381
	$RbNa_{12}$(f)	8.335	8.244	8.023	9.208	6.821	8.149
	$CsNa_{12}$(f)	8.310	8.223	7.997	9.180	6.807	8.123
	LiK_{12}(f)	6.571	6.511	6.295	7.552	5.309	6.465
	NaK_{12}(f)	6.417	6.334	6.138	7.399	5.133	6.322
	RbK_{12}(c)	6.163	6.094	5.886	7.150	4.927	6.081
	RbK_{12}(f)	6.222	6.135	5.946	7.209	4.945	6.134
	CsK_{12}(c)	6.051	6.017	5.778	7.039	4.880	5.969
	CsK_{12}(f)	6.186	6.099	5.910	7.171	4.915	6.098
	$LiRb_{12}$(f)	5.909	5.890	5.642	6.888	4.759	5.807
	$NaRb_{12}$(f)	5.791	5.749	5.524	6.767	4.614	5.699
	KRb_{12}(c)	5.711	5.643	5.446	6.686	4.488	5.620
	KRb_{12}(f)	5.654	5.604	5.388	6.630	4.474	5.566
	$CsRb_{12}$(c)	5.507	5.484	5.240	6.483	4.397	5.424
	$CsRb_{12}$(f)	5.569	5.520	5.304	6.541	4.398	5.481
	$LiCs_{12}$(f)	5.370	5.377	5.108	6.358	4.354	5.285
	$NaCs_{12}$(f)	5.266	5.247	5.003	6.248	4.217	5.193
	KCs_{12}(c)	5.137	5.058	4.882	6.090	3.896	4.977
	KCs_{12}(f)	5.114	5.082	4.849	6.075	4.048	5.039

	$RbCs_{12}$(c)	5.071	5.009	4.811	6.026	3.812	4.874
	$RbCs_{12}$(f)	5.064	5.032	4.799	6.021	4.001	4.988

eV) .

					Effect of basis set increment				
					Density Functional				
	RANGE-SE	DOUBLE HYBRID GGA			GGA			META-GGA	HYBRID GG
B3PW91	LC-ωPBE	B2PLYP		Systems	PBE	BP86	BPBE	TPSS	B3LYP
12.061	11.639	10.871		Li_{13}	1.007	0.857	1.029	0.963	0.791
8.216	7.829	7.500		Na_{13}	0.862	0.779	0.881	0.858	0.797
6.043	5.897	5.552		K_{13}	0.732	0.630	0.758	0.699	0.561
5.401	5.255	4.980		Rb_{13}	0.052	0.047	0.060	0.064	0.033
4.834	4.733	4.470		Cs_{13}	0.053	0.067	0.054	0.065	0.074
10.996	10.520	9.997		$NaLi_{12}$(c)	1.090	0.947	1.099	1.020	0.905
11.743	11.370	10.577		$NaLi_{12}$(f)	1.010	0.867	1.032	0.973	0.810
9.738	9.260	8.941		KLi_{12}(c)	1.032	0.899	1.047	0.956	0.859
11.555	11.182	10.405		KLi_{12}(f)	1.012	0.867	1.034	0.970	0.800
9.068	8.609	8.332		$RbLi_{12}$(c)	1.321	1.187	1.348	1.264	1.119
11.493	11.124	10.351		$RbLi_{12}$(f)	0.876	0.738	0.896	0.838	0.683
11.483	11.105	10.344		$CsLi_{12}$(f)	0.870	0.729	0.891	0.835	0.669
8.557	8.221	7.728		$LiNa_{12}$(c)	0.974	0.890	1.000	0.986	0.912
8.436	8.039	7.704		$LiNa_{12}$(f)	0.889	0.802	0.907	0.882	0.814
7.518	7.178	6.941		KNa_{12}(c)	0.768	0.681	0.788	0.768	0.705
8.044	7.683	7.336		KNa_{12}(f)	0.869	0.784	0.889	0.860	0.794
7.248	6.915	6.701		$RbNa_{12}$(c)	1.062	0.992	1.095	1.072	1.005
7.992	7.632	7.285		$RbNa_{12}$(f)	0.730	0.647	0.744	0.721	0.665
7.968	7.610	7.266		$CsNa_{12}$(f)	0.728	0.641	0.743	0.721	0.656
6.334	6.165	5.779		LiK_{12}(f)	0.746	0.642	0.771	0.707	0.573
6.181	6.049	5.632		NaK_{12}(f)	0.750	0.652	0.777	0.721	0.592
5.947	5.818	5.508		RbK_{12}(c)	0.823	0.741	0.848	0.795	0.687
5.992	5.856	5.499		RbK_{12}(f)	0.757	0.666	0.782	0.720	0.606
5.855	5.713	5.469		CsK_{12}(c)	0.886	0.800	0.914	0.860	0.745
5.956	5.830	5.453		CsK_{12}(f)	0.714	0.620	0.739	0.677	0.555
5.701	5.580	5.154		$LiRb_{12}$(f)	0.029	0.013	0.022	0.000	0.008
5.585	5.494	5.043		$NaRb_{12}$(f)	0.001	0.009	0.009	0.021	0.002
5.495	5.354	5.010		KRb_{12}(c)	0.105	0.122	0.100	0.071	0.154
5.449	5.316	5.019		KRb_{12}(f)	0.054	0.052	0.050	0.039	0.059
5.322	5.182	4.968		$CsRb_{12}$(c)	0.052	0.050	0.059	0.057	0.041
5.365	5.237	4.925		$CsRb_{12}$(f)	0.049	0.045	0.056	0.062	0.034
5.191	5.174	4.617		$LiCs_{12}$(f)	0.004	0.033	0.004	0.040	0.066
5.089	5.098	4.522		$NaCs_{12}$(f)	0.013	0.040	0.014	0.043	0.058
4.856	4.700	4.411		KCs_{12}(c)	0.106	0.100	0.104	0.070	0.055
4.933	4.815	4.422		KCs_{12}(f)	0.026	0.003	0.027	0.004	0.013

4.757	4.581	4.351	$RbCs_{12}$(c)	0.064	0.080	0.067	0.085	0.071
4.883	4.771	4.397	$RbCs_{12}$(f)	0.050	0.062	0.051	0.063	0.069

		PBE	BP86	BPBE	TPSS	B3LYP
**Li-K**	**Minimun**	0.714	0.620	0.739	0.677	0.555
	**Maximun**	1.321	1.187	1.348	1.264	1.119
	**Difference**	0.607	0.567	0.609	0.587	0.564
**Rb-Cs**	**Minimun**	0.001	0.003	0.004	0.000	0.002
	**Maximun**	0.106	0.122	0.104	0.085	0.154
	**Difference**	0.105	0.119	0.100	0.085	0.152

GA		RANGE-SE	DOUBLE HYBRID GGA
PBE0	B3PW91	LC-ωPBE	B2PLYP
0.826	0.842	0.495	0.651
0.789	0.801	0.641	0.575
0.651	0.653	0.473	0.339
0.046	0.049	0.037	0.133
0.054	0.059	0.059	0.125
0.917	0.928	0.600	0.767
0.839	0.855	0.538	0.620
0.867	0.884	0.580	0.692
0.838	0.852	0.521	0.614
1.166	1.182	0.891	0.879
0.717	0.729	0.461	0.469
0.711	0.722	0.444	0.478
0.895	0.913	0.734	0.656
0.809	0.821	0.654	0.585
0.708	0.717	0.594	0.493
0.791	0.804	0.631	0.571
0.987	1.012	0.839	0.752
0.664	0.672	0.530	0.461
0.662	0.669	0.524	0.457
0.659	0.661	0.467	0.367
0.669	0.673	0.490	0.359
0.718	0.730	0.475	0.462
0.676	0.681	0.494	0.377
0.785	0.798	0.550	0.514
0.635	0.638	0.450	0.351
0.023	0.018	0.029	0.004
0.002	0.006	0.033	0.044
0.103	0.110	0.093	0.033
0.051	0.050	0.040	0.069
0.048	0.052	0.046	0.142
0.044	0.046	0.039	0.122
0.015	0.022	0.087	0.077
0.025	0.030	0.079	0.081
0.058	0.065	0.012	0.010
0.014	0.010	0.001	0.067

0.058	0.051	0.060	0.127			
0.051	0.055	0.056	0.103			

PBE0	B3PW91	LC-ωPBE	B2PLYP			
0.635	0.638	0.444	0.339	**Minimun**	0.339	
1.166	1.182	0.891	0.879	**Maximun**	1.348	
0.531	0.544	0.447	0.539	**Minimun**	0.447	0.162
				**Maximun**	0.609	
0.002	0.006	0.001	0.004	**Minimun**	0.000	
0.103	0.110	0.093	0.142	**Maximun**	0.154	
0.101	0.104	0.091	0.139	**Minimun**	0.085	0.067
				**Maximun**	0.152	

Table S7. Atomization energies calculated with PBE and PBE0 functionals plus D3-BJ empirical dispersi

	Density Functional				
	GGA		HYBRID GGA		DMC*
Systems	PBE	PBE(QZ)	PBE0	PBE0(QZ)	REFERENCE
Li_{13}	13.039	13.260	12.859	13.071	12.937
Na_{13}	9.246	8.648	9.126	8.600	8.954
K_{13}	6.811	6.762	6.824	6.799	7.048
Rb_{13}	6.117	6.131	6.146	6.160	6.662
Cs_{13}	5.442	5.492	5.483	5.536	6.056
$NaLi_{12}$(c)	11.939	12.099	11.760	11.909	11.978
$NaLi_{12}$(f)	12.727	12.896	12.552	12.720	12.669
KLi_{12}(c)	10.624	10.872	10.461	10.700	10.99
KLi_{12}(f)	12.524	12.724	12.355	12.549	12.493
$RbLi_{12}$(c)	9.898	10.031	9.750	9.865	10.389
$RbLi_{12}$(f)	12.457	12.653	12.290	12.478	12.398
$CsLi_{12}$(f)	12.444	12.655	12.274	12.479	12.496
$LiNa_{12}$(c)	9.597	9.163	9.481	9.099	9.313
$LiNa_{12}$(f)	9.461	8.937	9.334	8.861	9.208
KNa_{12}(c)	8.467	8.055	8.380	8.013	8.433
KNa_{12}(f)	9.053	8.525	8.945	8.467	8.709
$RbNa_{12}$(c)	8.158	7.679	8.079	7.634	8.207
$RbNa_{12}$(f)	8.995	8.464	8.888	8.407	8.775
$CsNa_{12}$(f)	8.964	8.459	8.857	8.402	8.739
LiK_{12}(f)	7.114	7.081	7.106	7.095	7.379
NaK_{12}(f)	6.961	6.888	6.967	6.920	7.227
RbK_{12}(c)	6.690	6.627	6.712	6.662	6.969
RbK_{12}(f)	6.755	6.708	6.770	6.744	7.004
CsK_{12}(c)	6.575	6.512	6.599	6.548	6.761
CsK_{12}(f)	6.712	6.672	6.728	6.709	6.896
$LiRb_{12}$(f)	6.426	6.447	6.437	6.454	6.958
$NaRb_{12}$(f)	6.311	6.293	6.335	6.319	6.839
KRb_{12}(c)	6.235	6.243	6.262	6.272	6.699
KRb_{12}(f)	6.169	6.177	6.197	6.206	6.703
$CsRb_{12}$(c)	6.003	6.018	6.038	6.053	6.673
$CsRb_{12}$(f)	6.075	6.092	6.104	6.121	6.642
$LiCs_{12}$(f)	5.807	5.868	5.835	5.900	6.324
$NaCs_{12}$(f)	5.706	5.734	5.748	5.784	6.225
KCs_{12}(c)	5.582	5.627	5.538	5.664	6.007
KCs_{12}(f)	5.549	5.591	5.590	5.636	5.983
$RbCs_{12}$(c)	5.508	5.560	5.428	5.487	6.042
$RbCs_{12}$(f)	5.497	5.546	5.538	5.590	6.111

*Diffusion Monte Carlo

	Density Functional
	GGA

	PBE	PBE(QZ)	PBE0	PBE0(QZ)
**SD**	2.424	2.456	2.348	2.377

**Absolute deviations**				
	**Density Functional**			
	**GGA**			
**Systems**	**PBE**	**PBE(QZ)**	**PBE0**	**PBE0(QZ)**
Li_{13}	0.102	0.323	0.078	0.134
Na_{13}	0.292	0.306	0.172	0.354
K_{13}	0.237	0.286	0.224	0.249
Rb_{13}	0.545	0.531	0.516	0.502
Cs_{13}	0.614	0.564	0.573	0.520
$NaLi_{12}$(c)	0.039	0.121	0.218	0.069
$NaLi_{12}$(f)	0.058	0.227	0.117	0.051
KLi_{12}(c)	0.366	0.118	0.529	0.290
KLi_{12}(f)	0.031	0.231	0.138	0.056
$RbLi_{12}$(c)	0.491	0.358	0.639	0.524
$RbLi_{12}$(f)	0.059	0.255	0.108	0.080
$CsLi_{12}$(f)	0.052	0.159	0.222	0.017
$LiNa_{12}$(c)	0.284	0.150	0.168	0.214
$LiNa_{12}$(f)	0.253	0.271	0.126	0.347
KNa_{12}(c)	0.034	0.378	0.053	0.420
KNa_{12}(f)	0.344	0.184	0.236	0.242
$RbNa_{12}$(c)	0.049	0.528	0.128	0.573
$RbNa_{12}$(f)	0.220	0.311	0.113	0.368
$CsNa_{12}$(f)	0.225	0.280	0.118	0.337
LiK_{12}(f)	0.265	0.298	0.273	0.284
NaK_{12}(f)	0.266	0.339	0.260	0.307
RbK_{12}(c)	0.279	0.342	0.257	0.307
RbK_{12}(f)	0.249	0.296	0.234	0.260
CsK_{12}(c)	0.186	0.249	0.162	0.213
CsK_{12}(f)	0.184	0.224	0.168	0.187
$LiRb_{12}$(f)	0.532	0.511	0.521	0.504
$NaRb_{12}$(f)	0.528	0.546	0.504	0.520
KRb_{12}(c)	0.464	0.456	0.437	0.427
KRb_{12}(f)	0.534	0.526	0.506	0.497
$CsRb_{12}$(c)	0.670	0.655	0.635	0.620
$CsRb_{12}$(f)	0.567	0.550	0.538	0.521
$LiCs_{12}$(f)	0.517	0.456	0.489	0.424
$NaCs_{12}$(f)	0.519	0.491	0.477	0.441
KCs_{12}(c)	0.425	0.380	0.469	0.343
KCs_{12}(f)	0.434	0.392	0.393	0.347
$RbCs_{12}$(c)	0.534	0.482	0.614	0.555
$RbCs_{12}$(f)	0.614	0.565	0.573	0.521

	Density Functional			
	GGA			
	PBE	PBE(QZ)	PBE0	PBE0(QZ)
**MAD**	0.326	0.361	0.324	0.341
**MAE**	0.670	0.655	0.639	0.620
**SD(MAD)**	0.194	0.142	0.188	0.164

Signed deviations

	Density Functional			
	GGA			
**Systems**	**PBE**	**PBE(QZ)**	**PBE0**	**PBE0(QZ)**
Li_{13}	0.102	0.323	-0.078	0.134
Na_{13}	0.292	-0.306	0.172	-0.354
K_{13}	-0.237	-0.286	-0.224	-0.249
Rb_{13}	-0.545	-0.531	-0.516	-0.502
Cs_{13}	-0.614	-0.564	-0.573	-0.520
$NaLi_{12}$(c)	-0.039	0.121	-0.218	-0.069
$NaLi_{12}$(f)	0.058	0.227	-0.117	0.051
KLi_{12}(c)	-0.366	-0.118	-0.529	-0.290
KLi_{12}(f)	0.031	0.231	-0.138	0.056
$RbLi_{12}$(c)	-0.491	-0.358	-0.639	-0.524
$RbLi_{12}$(f)	0.059	0.255	-0.108	0.080
$CsLi_{12}$(f)	-0.052	0.159	-0.222	-0.017
$LiNa_{12}$(c)	0.284	-0.150	0.168	-0.214
$LiNa_{12}$(f)	0.253	-0.271	0.126	-0.347
KNa_{12}(c)	0.034	-0.378	-0.053	-0.420
KNa_{12}(f)	0.344	-0.184	0.236	-0.242
$RbNa_{12}$(c)	-0.049	-0.528	-0.128	-0.573
$RbNa_{12}$(f)	0.220	-0.311	0.113	-0.368
$CsNa_{12}$(f)	0.225	-0.280	0.118	-0.337
LiK_{12}(f)	-0.265	-0.298	-0.273	-0.284
NaK_{12}(f)	-0.266	-0.339	-0.260	-0.307
RbK_{12}(c)	-0.279	-0.342	-0.257	-0.307
RbK_{12}(f)	-0.249	-0.296	-0.234	-0.260
CsK_{12}(c)	-0.186	-0.249	-0.162	-0.213
CsK_{12}(f)	-0.184	-0.224	-0.168	-0.187
$LiRb_{12}$(f)	-0.532	-0.511	-0.521	-0.504
$NaRb_{12}$(f)	-0.528	-0.546	-0.504	-0.520
KRb_{12}(c)	-0.464	-0.456	-0.437	-0.427
KRb_{12}(f)	-0.534	-0.526	-0.506	-0.497
$CsRb_{12}$(c)	-0.670	-0.655	-0.635	-0.620
$CsRb_{12}$(f)	-0.567	-0.550	-0.538	-0.521

$LiCs_{12}$(f)	-0.517	-0.456	-0.489	-0.424
$NaCs_{12}$(f)	-0.519	-0.491	-0.477	-0.441
KCs_{12}(c)	-0.425	-0.380	-0.469	-0.343
KCs_{12}(f)	-0.434	-0.392	-0.393	-0.347
$RbCs_{12}$(c)	-0.534	-0.482	-0.614	-0.555
$RbCs_{12}$(f)	-0.614	-0.565	-0.573	-0.521

	**Density Functional**			
	**GGA**			
	**PBE**	**PBE(QZ)**	**PBE0**	**PBE0(QZ)**
**MSD**	-0.223	-0.289	-0.274	-0.324
**SD(MSD)**	0.307	0.258	0.256	0.196

on corrections, with the def2-TZVP and def2-QZVPPD basis sets, alon[illegible]

[illegible] (DMC) data from reference *Chem. Phys.* **2023**, *565* , 111767.

Table S8. Atomization energies calculated with PBE functional plus D3 and D3-BJ empirical dispersion c

*Diffusion Monte Carlo

	Density Functional				
	GGA				DMC*
Systems	PBE(D3-BJ	PBE(D3)	PBE(D4)	PBE(VV10)	REFERENCE
Li_{13}	13.039	12.632	12.710	12.841	12.937
Na_{13}	9.246	9.273	8.954	9.031	8.954
K_{13}	6.811	6.952	6.618	6.738	7.048
Rb_{13}	6.117	6.322	5.948	6.048	6.662
Cs_{13}	5.442	5.638	5.341	5.459	6.056
$NaLi_{12}$(c)	11.939	11.594	11.611	11.763	11.978
$NaLi_{12}$(f)	12.727	12.341	12.393	12.528	12.669
KLi_{12}(c)	10.624	10.301	10.316	10.454	10.99
KLi_{12}(f)	12.524	12.152	12.199	12.333	12.493
$RbLi_{12}$(c)	9.898	9.646	9.624	9.781	10.389
$RbLi_{12}$(f)	12.457	12.090	12.136	12.272	12.398
$CsLi_{12}$(f)	12.444	12.079	12.127	12.264	12.496
$LiNa_{12}$(c)	9.597	9.554	9.317	9.387	9.313
$LiNa_{12}$(f)	9.461	9.444	9.166	9.247	9.208
KNa_{12}(c)	8.467	8.542	8.197	8.280	8.433
KNa_{12}(f)	9.053	9.103	8.766	8.853	8.709
$RbNa_{12}$(c)	8.158	8.236	7.906	7.989	8.207
$RbNa_{12}$(f)	8.995	9.053	8.713	8.799	8.775
$CsNa_{12}$(f)	8.964	9.020	8.686	8.776	8.739
LiK_{12}(f)	7.114	7.217	6.919	7.029	7.379
NaK_{12}(f)	6.961	7.100	6.768	6.878	7.227
RbK_{12}(c)	6.690	6.822	6.504	6.617	6.969
RbK_{12}(f)	6.755	6.899	6.567	6.684	7.004
CsK_{12}(c)	6.575	6.668	6.354	6.499	6.761
CsK_{12}(f)	6.712	6.855	6.525	6.648	6.896
$LiRb_{12}$(f)	6.426	6.578	6.250	6.350	6.958
$NaRb_{12}$(f)	6.311	6.503	6.136	6.234	6.839
KRb_{12}(c)	6.235	6.436	6.107	6.162	6.699
KRb_{12}(f)	6.169	6.366	5.993	6.099	6.703
$CsRb_{12}$(c)	6.003	6.184	5.857	5.948	6.673
$CsRb_{12}$(f)	6.075	6.279	5.908	6.012	6.642
$LiCs_{12}$(f)	5.807	5.953	5.674	5.810	6.324
$NaCs_{12}$(f)	5.706	5.887	5.574	5.710	6.225
KCs_{12}(c)	5.582	5.774	5.466	5.591	6.007
KCs_{12}(f)	5.549	5.734	5.442	5.559	5.983
$RbCs_{12}$(c)	5.508	5.717	5.406	5.521	6.042
$RbCs_{12}$(f)	5.497	5.692	5.396	5.509	6.111

	Density Functional
	GGA

	PBE(D3-BJ	PBE(D3)	PBE(D4)	PBE(VV10)
**SD**	2.424	2.224	2.352	2.359

Absolute deviations

	**Density Functional**			
	**GGA**			
**Systems**	**PBE(D3-BJ**	**PBE(D3)**	**PBE(D4)**	**PBE(VV10)**
Li_{13}	0.102	0.305	0.227	0.096
Na_{13}	0.292	0.319	0.000	0.077
K_{13}	0.237	0.096	0.430	0.310
Rb_{13}	0.545	0.340	0.714	0.614
Cs_{13}	0.614	0.418	0.715	0.597
$NaLi_{12}$(c)	0.039	0.384	0.367	0.215
$NaLi_{12}$(f)	0.058	0.328	0.276	0.141
KLi_{12}(c)	0.366	0.689	0.674	0.536
KLi_{12}(f)	0.031	0.341	0.294	0.160
$RbLi_{12}$(c)	0.491	0.743	0.765	0.608
$RbLi_{12}$(f)	0.059	0.308	0.262	0.126
$CsLi_{12}$(f)	0.052	0.417	0.369	0.232
$LiNa_{12}$(c)	0.284	0.241	0.004	0.074
$LiNa_{12}$(f)	0.253	0.236	0.042	0.039
KNa_{12}(c)	0.034	0.109	0.236	0.153
KNa_{12}(f)	0.344	0.394	0.057	0.144
$RbNa_{12}$(c)	0.049	0.029	0.301	0.218
$RbNa_{12}$(f)	0.220	0.278	0.062	0.024
$CsNa_{12}$(f)	0.225	0.281	0.053	0.037
LiK_{12}(f)	0.265	0.162	0.460	0.350
NaK_{12}(f)	0.266	0.127	0.459	0.349
RbK_{12}(c)	0.279	0.147	0.465	0.352
RbK_{12}(f)	0.249	0.105	0.437	0.320
CsK_{12}(c)	0.186	0.093	0.407	0.262
CsK_{12}(f)	0.184	0.041	0.371	0.248
$LiRb_{12}$(f)	0.532	0.380	0.708	0.608
$NaRb_{12}$(f)	0.528	0.336	0.703	0.605
KRb_{12}(c)	0.464	0.263	0.592	0.537
KRb_{12}(f)	0.534	0.337	0.710	0.604
$CsRb_{12}$(c)	0.670	0.489	0.816	0.725
$CsRb_{12}$(f)	0.567	0.363	0.734	0.630
$LiCs_{12}$(f)	0.517	0.371	0.650	0.514
$NaCs_{12}$(f)	0.519	0.338	0.651	0.515
KCs_{12}(c)	0.425	0.233	0.541	0.416
KCs_{12}(f)	0.434	0.249	0.541	0.424
$RbCs_{12}$(c)	0.534	0.325	0.636	0.521
$RbCs_{12}$(f)	0.614	0.419	0.715	0.602

	Density Functional			
	GGA			
	PBE(D3-BJ	PBE(D3)	PBE(D4)	PBE(VV10)
**MAD**	0.326	0.298	0.444	0.351
**MAE**	0.670	0.743	0.816	0.725
**SD(MAD)**	0.194	0.152	0.243	0.210

Signed deviations

	Density Functional			
	GGA			
**Systems**	PBE(D3-BJ	PBE(D3)	PBE(D4)	PBE(VV10)
Li_{13}	0.102	-0.305	-0.227	-0.096
Na_{13}	0.292	0.319	0.000	0.077
K_{13}	-0.237	-0.096	-0.430	-0.310
Rb_{13}	-0.545	-0.340	-0.714	-0.614
Cs_{13}	-0.614	-0.418	-0.715	-0.597
$NaLi_{12}$(c)	-0.039	-0.384	-0.367	-0.215
$NaLi_{12}$(f)	0.058	-0.328	-0.276	-0.141
KLi_{12}(c)	-0.366	-0.689	-0.674	-0.536
KLi_{12}(f)	0.031	-0.341	-0.294	-0.160
$RbLi_{12}$(c)	-0.491	-0.743	-0.765	-0.608
$RbLi_{12}$(f)	0.059	-0.308	-0.262	-0.126
$CsLi_{12}$(f)	-0.052	-0.417	-0.369	-0.232
$LiNa_{12}$(c)	0.284	0.241	0.004	0.074
$LiNa_{12}$(f)	0.253	0.236	-0.042	0.039
KNa_{12}(c)	0.034	0.109	-0.236	-0.153
KNa_{12}(f)	0.344	0.394	0.057	0.144
$RbNa_{12}$(c)	-0.049	0.029	-0.301	-0.218
$RbNa_{12}$(f)	0.220	0.278	-0.062	0.024
$CsNa_{12}$(f)	0.225	0.281	-0.053	0.037
LiK_{12}(f)	-0.265	-0.162	-0.460	-0.350
NaK_{12}(f)	-0.266	-0.127	-0.459	-0.349
RbK_{12}(c)	-0.279	-0.147	-0.465	-0.352
RbK_{12}(f)	-0.249	-0.105	-0.437	-0.320
CsK_{12}(c)	-0.186	-0.093	-0.407	-0.262
CsK_{12}(f)	-0.184	-0.041	-0.371	-0.248
$LiRb_{12}$(f)	-0.532	-0.380	-0.708	-0.608
$NaRb_{12}$(f)	-0.528	-0.336	-0.703	-0.605
KRb_{12}(c)	-0.464	-0.263	-0.592	-0.537
KRb_{12}(f)	-0.534	-0.337	-0.710	-0.604
$CsRb_{12}$(c)	-0.670	-0.489	-0.816	-0.725
$CsRb_{12}$(f)	-0.567	-0.363	-0.734	-0.630

$LiCs_{12}$(f)	-0.517	-0.371	-0.650	-0.514
$NaCs_{12}$(f)	-0.519	-0.338	-0.651	-0.515
KCs_{12}(c)	-0.425	-0.233	-0.541	-0.416
KCs_{12}(f)	-0.434	-0.249	-0.541	-0.424
$RbCs_{12}$(c)	-0.534	-0.325	-0.636	-0.521
$RbCs_{12}$(f)	-0.614	-0.419	-0.715	-0.602

	**Density Functional**			
	**GGA**			
	**PBE(D3-BJ**	**PBE(D3)**	**PBE(D4)**	**PBE(VV10)**
**MSD**	-0.223	-0.196	-0.441	-0.330
**SD(MSD)**	0.307	0.271	0.249	0.242

:orrections, with the def2-TZVP basis set, along with Mean Absolute D

› (DMC) data from reference Chem. Phys. 2023, 565, 111767.

Table S9. Atomization energies per atom calculated with PBE plus D3-BJ empirical dispersion correc

Cluster	Structure	Multiplicity	def2-TZVPP	def2-QZVPP	cc-pVTZ	cc-pVQZ	aug-cc-pVTZ
			**PBE**				
Li_4	Sym-Linear	1	478.16	480.62	486.03	489.24	487.97
		3	391.07	400.37	405.14	407.89	412.35
	Linear	1	479.85	484.17	488.69	492.55	490.82
		3	390.39	398.98	403.93	406.34	406.25
	Rhomboid	1	658.83	673.99	678.35	682.73	680.43
		3	582.45	602.10	606.77	610.46	608.60
Li_5	Linear	2	490.04	493.81	499.35	502.56	501.60
		4	358.25	368.68	373.18	375.53	376.10
	W	2	714.33	730.33	735.69	739.39	737.55
		4	521.78	548.12	553.42	558.01	555.52
	TBP	2	748.39	766.61	771.69	776.87	773.68
		4	725.39	748.85	753.85	759.22	755.59

Cluster	Structure	Multiplicity	def2-TZVPP	def2-QZVPP	cc-pVTZ	cc-pVQZ	aug-cc-pVTZ
				**Absolute deviation (Calc-Ref)**			
Li_4	Sym-Linear	1	40.84	38.38	32.97	29.76	31.03
		3	4.07	13.37	18.14	20.89	25.35
	Linear	1	55.15	50.83	46.31	42.45	44.18
		3	2.39	10.98	15.93	18.34	18.25
	Rhomboid	1	94.17	79.01	74.65	70.27	72.57
		3	38.55	18.90	14.23	10.54	12.40
Li_5	Linear	2	20.96	17.19	11.65	8.44	9.40
		4	15.25	25.68	30.18	32.53	33.10
	W	2	57.67	41.67	36.31	32.61	34.45
		4	22.22	4.12	9.42	14.01	11.52
	TBP	2	52.61	34.39	29.31	24.13	27.32
		4	28.61	5.15	0.15	5.22	1.59

	def2-TZVPP	def2-QZVPP	cc-pVTZ	cc-pVQZ	aug-cc-pVTZ
**MAD**	36.04	28.30	26.60	25.77	26.76

Cluster	Structure	Multiplicity	def2-TZVPP	def2-QZVPP	cc-pVTZ	cc-pVQZ	aug-cc-pVTZ
				**Signed Deviation (Calc-Ref)**			
Li_4	Sym-Linear	1	-40.84	-38.38	-32.97	-29.76	-31.03
		3	4.07	13.37	18.14	20.89	25.35
	Linear	1	-55.15	-50.83	-46.31	-42.45	-44.18
		3	2.39	10.98	15.93	18.34	18.25
	Rhomboid	1	-94.17	-79.01	-74.65	-70.27	-72.57
		3	-38.55	-18.90	-14.23	-10.54	-12.40
Li_5	Linear	2	-20.96	-17.19	-11.65	-8.44	-9.40
		4	15.25	25.68	30.18	32.53	33.10
	W	2	-57.67	-41.67	-36.31	-32.61	-34.45

	4	-22.22	4.12	9.42	14.01	11.52
TBP	2	-52.61	-34.39	-29.31	-24.13	-27.32
	4	-28.61	-5.15	-0.15	5.22	1.59

	def2-TZVPP	def2-QZVPP	cc-pVTZ	cc-pVQZ	aug-cc-pVTZ
**MSD**	-32.42	-19.28	-14.33	-10.60	-11.80

ctions and different basis sets along with Mean Absolute Deviations (MADs) and Mean Signed Deviations (MSD

* W3X-L data from reference *J. Chem. Theory*

	REFERENCE(W3X-L)*
aug-cc-pVQZ	Composite
490.98	519
410.85	387
493.98	535
409.17	388
684.78	753
613.10	621
504.55	511
378.79	343
741.98	772
560.43	544
778.65	801
761.20	754

aug-cc-pVQZ
28.02
23.85
41.02
21.17
68.22
7.90
6.45
35.79
30.02
16.43
22.35
7.20

aug-cc-pVQZ
25.70

aug-cc-pVQZ
-28.02
23.85
-41.02
21.17
-68.22
-7.90
-6.45
35.79
-30.02

16.43
-22.35
7.20

**aug-cc-pVQZ**
-8.29

s) compared to W3X-L reference data (in meV)

Comput. **2024**, *20* , 10491–10506.

SUPPORTING INFORMATION

Atomization Energy Calculations in 13-Atom Alkali Metal Clusters: Is there an Appropriate Exchange-Correlation Functional?

Wagner F. D. Angelotti,[a] Lucila C. Z. Angelotti,[b] Roberto L. A. Haiduke[c]

[a]Instituto de Ciências Tecnológicas e Exatas, Departamento de Matemática Aplicada, Universidade Federal do Triângulo Mineiro, Av. Dr. Randolfo Borges Jr., 1400, 38064-200, Uberaba, Minas Gerais, Brazil

[b]Centro Universitário Barão de Mauá, R. Ramos de Azevedo, 423, 14090-062, Ribeirão Preto, São Paulo, Brazil

[c]Departamento de Química e Física Molecular, Instituto de Química de São Carlos, Universidade de São Paulo, Av. Trabalhador São-Carlense, 400, CP 780, 13560-970, São Carlos, São Paulo, Brazil

Hierarchical and Non-Hierarchical Clustering of XC Functionals

In order to identify patterns in atomization energy values, cluster analysis was employed to group 32 XC functionals and reference data based on similarity values. The aim was to create more homogeneous clusters while maximizing inter-cluster differences. Following the methodology proposed by Hair et al.,[1] a hierarchical agglomerative technique was initially used to generate a complete set of solutions, detecting and eliminating outliers, and determining the optimal number of clusters.

This process yields a basic group structure with the fewest possible clusters while maintaining internal homogeneity. Subsequently, the resulting cluster centers serve as initial inputs for a non-hierarchical refinement stage.

Data standardization was performed to mitigate the impact of variable scales and magnitudes on clustering, as most techniques are sensitive to these factors. The centroid method and Euclidean distance were chosen for cluster formation due to their common use and robustness to outliers.[1-3]

Cluster validity was assessed through the cophenetic correlation, which yielded a value of 0.7715, indicating a strong correlation and confirming valid clustering.[4] The silhouette index was employed to determine the optimal number of clusters. A value of three (3) was selected based on the highest index (0.5372), suggesting appropriate cluster allocation.[5]

Initial clustering resulted in three groups:

- ***Cluster 1 (C1, n = 2)***: SVWN5, and TPSS;

- ***Cluster 2 (C2, n = 18)***: BLYP, OLYP, HCTH, τ-HCTH, B3LYP, X3LYP, BHANDHLYP, LC-BLYP, LC-PBEPBE, CAM-B3LYP, LC-ωPBE, ωB97X, LC-QTP, CAM-QTP00, CAM-QTP01, CAM-QTP02, B2PLYP, and MPW2PLYP;
- ***Cluster 3 (C3, n = 13)*****:** PBE, BP86, BPBE, PW91, PBE0, B3P86, B3PW91, MPW1PW91, M06-2X, BMK, PBEQIDH, PBE0DH, and REFERENCE data;

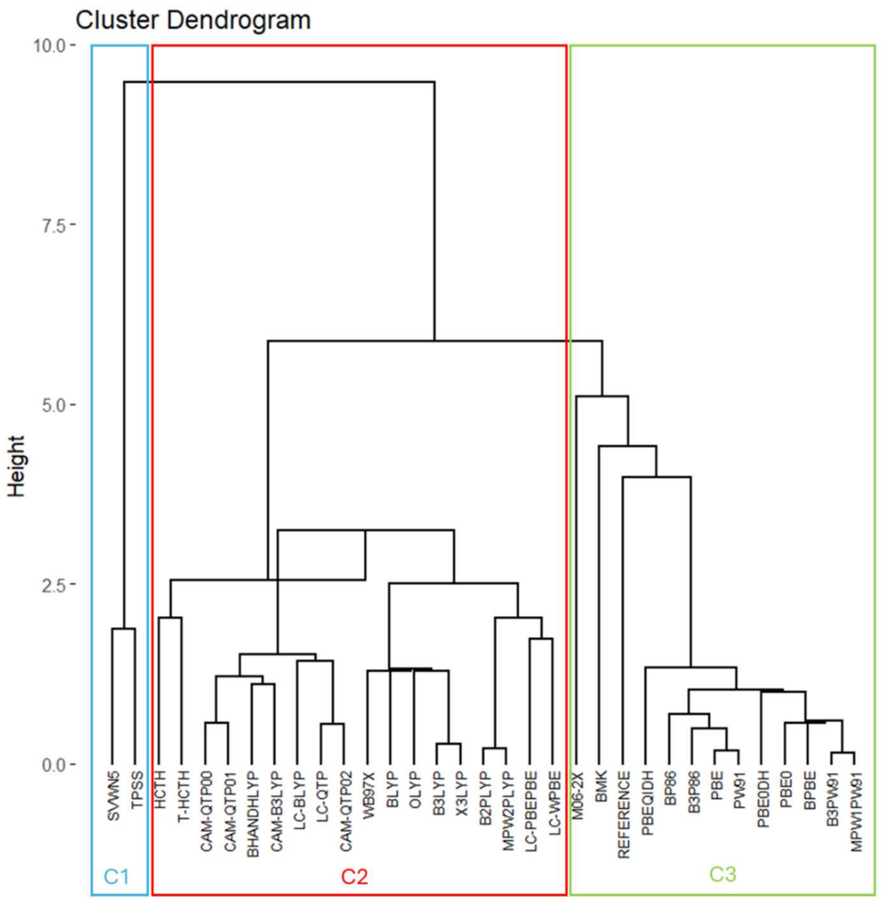

The final refinement stage utilized the K-means method with the previous cluster centers as initial centroids. This resulted in the following three clusters:

- ***Cluster 1 (n = 2)***: SVWN5, and TPSS;
- ***Cluster 2 (n = 15)***: PBE, BP86, BPBE, PW91, PBE0, B3P86, B3PW91, MPW1PW91, M06-2X, BMK, LC-PBEPBE, LC-ωPBE, PBEQIDH, PBE0DH, and REFERENCE data;
- ***Cluster 3 (n = 16)***: BLYP, OLYP, HCTH, τ-HCTH, B3LYP, X3LYP, BHANDHLYP, LC-BLYP, CAM-B3LYP, ωB97X, LC-QTP, CAM-QTP00, CAM-QTP01, CAM-QTP02, B2PLYP, and MPW2PLYP.

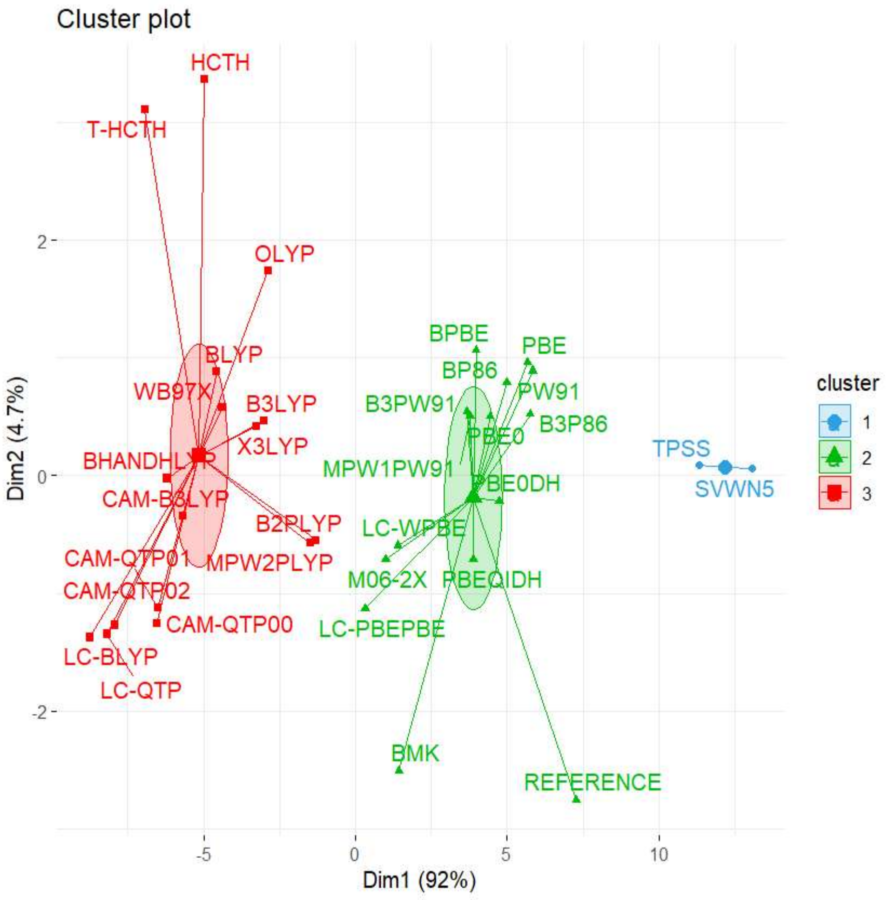

The R software[6] was employed to generate both the hierarchical and non-hierarchical clustering representations of the density functionals.

References

(1) Hair Jr, J. F.; Black, W. C.; Babin, B. J.; Anderson, R. E.; Tatham, R. L. Análise multivariada de dados. 6. ed. Porto Alegre: Bookman, 2009. 688 p.
(2) Mingoti, S. A. Análise de dados através de métodos de estatística multivariada: uma abordagem aplicada. Belo Horizonte: Editora UFMG, 2005.
(3) Lattin, J.; Carroll, J. D.; Green, P. E. Análise de dados multivariados. São Paulo: Cengage Learning, 2011. 455 p.
(4) Artes, R.; Barroso, L. P. Métodos multivariados de análise estatística. São Paulo: Blucher, 2023. 534 p.
(5) Shahapure, K. R.; Nicholas, C. Cluster Quality Analysis Using Silhouette Score. 2020 IEEE 7th International Conference on Data Science and Advanced Analytics (DSAA), Sydney, NSW, Australia, 2020, pp. 747-748, doi: 10.1109/DSAA49011.2020.00096.
(6) R Core Team. R: A Language and Environment for Statistical Computing. R Foundation for Statistical Computing, Vienna, Austria, 2024. <https://www.R-project.org/>. (accessed 2024–08–31).
