# Supplementary material for: Atomization Energy Calculations in 13‐Atom Alkali Metal Clusters: Is There an Appropriate Exchange‐Correlation Functional?
Source: J Comput Chem. 2025 Jul 21;46(20):e70187. doi: 10.1002/jcc.70187 (PMC12277945; doi:10.1002/jcc.70187)
Supplement: Supplementary file 1 — Data S1. Supporting Information. [file JCC-46-0-s001.zip › SI_dend_Km_rev.docx]

**SUPPORTING INFORMATION**

**Atomization Energy Calculations in 13-Atom Alkali Metal Clusters: Is there an Appropriate Exchange-Correlation Functional?**

Wagner F. D. Angelotti,^a^ Lucila C. Z. Angelotti,^b^ Roberto L. A. Haiduke^c^

^a^Instituto de Ciências Tecnológicas e Exatas, Departamento de Matemática Aplicada, Universidade Federal do Triângulo Mineiro, Av. Dr. Randolfo Borges Jr., 1400, 38064-200, Uberaba, Minas Gerais, Brazil

^b^Centro Universitário Barão de Mauá, R. Ramos de Azevedo, 423, 14090-062, Ribeirão Preto, São Paulo, Brazil

^c^Departamento de Química e Física Molecular, Instituto de Química de São Carlos, Universidade de São Paulo, Av. Trabalhador São-Carlense, 400, CP 780, 13560-970, São Carlos, São Paulo, Brazil

**Hierarchical and Non-Hierarchical Clustering of XC Functionals**

In order to identify patterns in atomization energy values, cluster analysis was employed to group 32 XC functionals and reference data based on similarity values. The aim was to create more homogeneous clusters while maximizing inter-cluster differences. Following the methodology proposed by Hair et al.,^1^ a hierarchical agglomerative technique was initially used to generate a complete set of solutions, detecting and eliminating outliers, and determining the optimal number of clusters.

This process yields a basic group structure with the fewest possible clusters while maintaining internal homogeneity. Subsequently, the resulting cluster centers serve as initial inputs for a non-hierarchical refinement stage.

Data standardization was performed to mitigate the impact of variable scales and magnitudes on clustering, as most techniques are sensitive to these factors. The centroid method and Euclidean distance were chosen for cluster formation due to their common use and robustness to outliers.^1-3^

Cluster validity was assessed through the cophenetic correlation, which yielded a value of 0.7715, indicating a strong correlation and confirming valid clustering.^4^ The silhouette index was employed to determine the optimal number of clusters. A value of three (3) was selected based on the highest index (0.5372), suggesting appropriate cluster allocation.^5^

Initial clustering resulted in three groups:

- ***Cluster 1 (C1, n = 2)***: SVWN5, and TPSS;
- ***Cluster 2 (C2, n = 18)***: BLYP, OLYP, HCTH, τ-HCTH, B3LYP, X3LYP, BHANDHLYP, LC-BLYP, LC-PBEPBE, CAM-B3LYP, LC-ωPBE, ωB97X, LC-QTP, CAM-QTP00, CAM-QTP01, CAM-QTP02, B2PLYP, and MPW2PLYP;
- ***Cluster 3 (C3, n = 13)*:** PBE, BP86, BPBE, PW91, PBE0, B3P86, B3PW91, MPW1PW91, M06-2X, BMK, PBEQIDH, PBE0DH, and REFERENCE data;


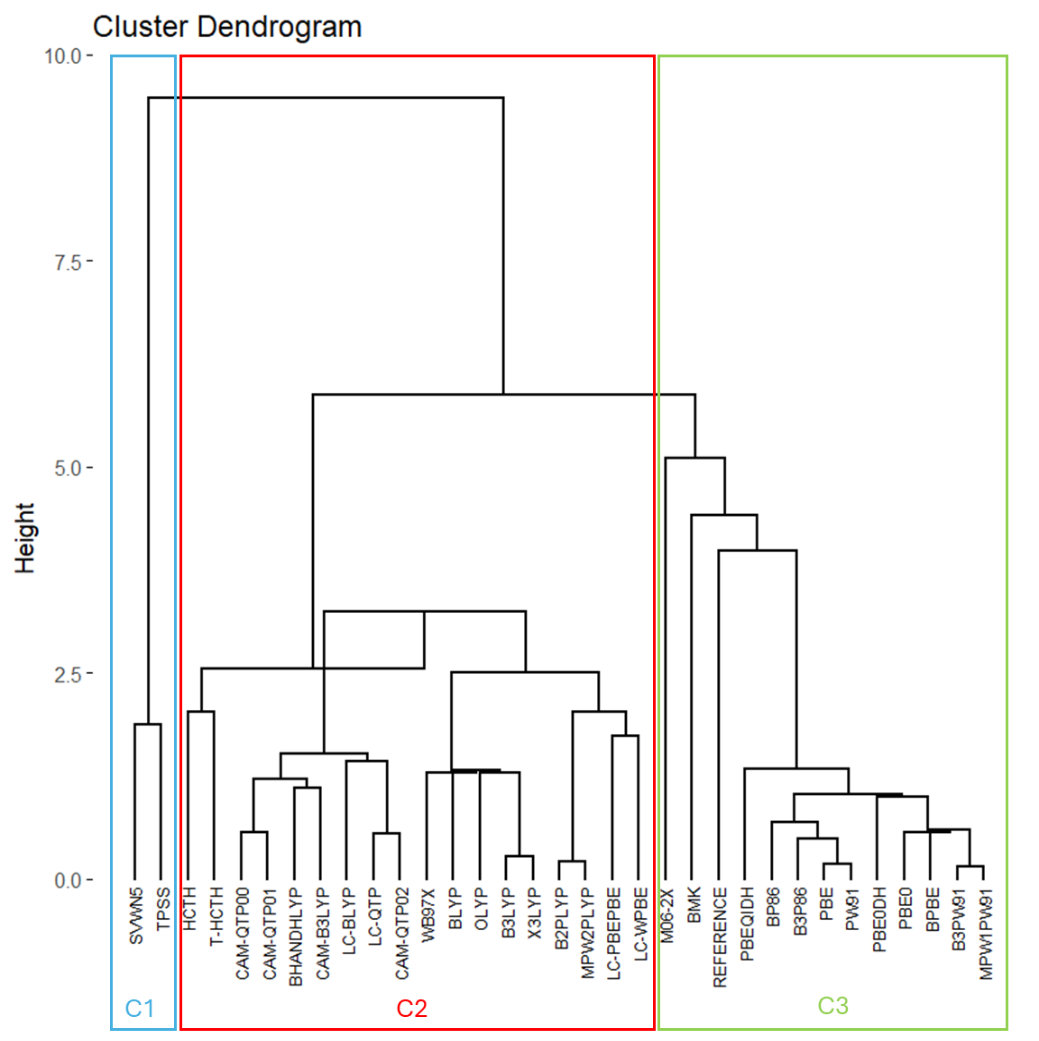


The final refinement stage utilized the K-means method with the previous cluster centers as initial centroids. This resulted in the following three clusters:

- ***Cluster 1 (n = 2)***: SVWN5, and TPSS;
- ***Cluster 2 (n = 15)***: PBE, BP86, BPBE, PW91, PBE0, B3P86, B3PW91, MPW1PW91, M06-2X, BMK, LC-PBEPBE, LC-ωPBE, PBEQIDH, PBE0DH, and REFERENCE data;
- ***Cluster 3 (n = 16)***: BLYP, OLYP, HCTH, τ-HCTH, B3LYP, X3LYP, BHANDHLYP, LC-BLYP, CAM-B3LYP, ωB97X, LC-QTP, CAM-QTP00, CAM-QTP01, CAM-QTP02, B2PLYP, and MPW2PLYP.


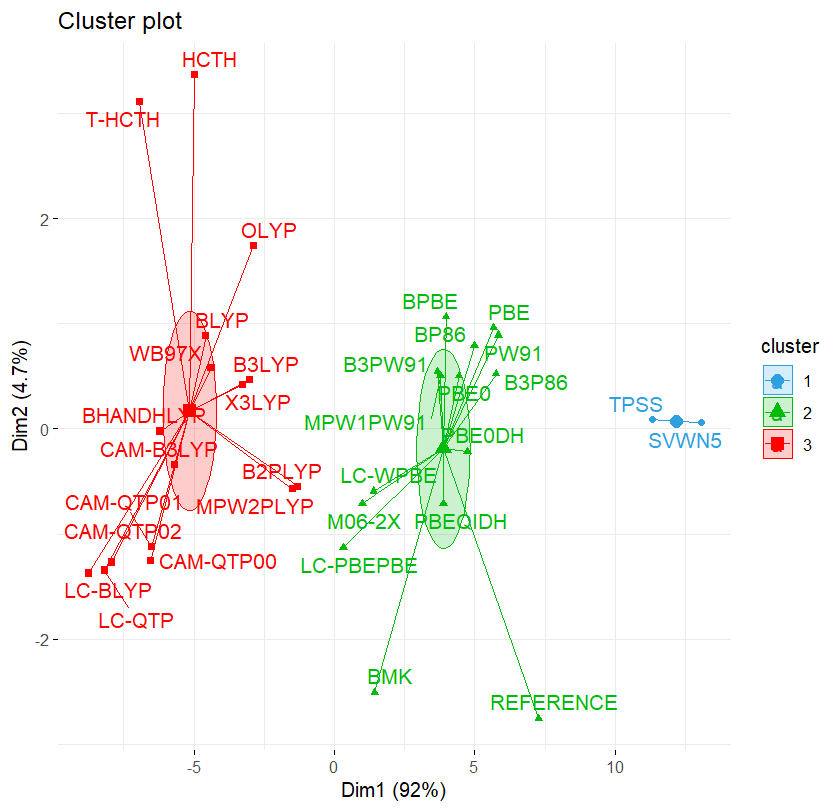


The R software^6^ was employed to generate both the hierarchical and non-hierarchical clustering representations of the density functionals.

**References**

1. Hair Jr, J. F.; Black, W. C.; Babin, B. J.; Anderson, R. E.; Tatham, R. L. Análise multivariada de dados. 6. ed. Porto Alegre: Bookman, 2009. 688 p.
2. Mingoti, S. A. Análise de dados através de métodos de estatística multivariada: uma abordagem aplicada. Belo Horizonte: Editora UFMG, 2005.
3. Lattin, J.; Carroll, J. D.; Green, P. E. Análise de dados multivariados. São Paulo: Cengage Learning, 2011. 455 p.
4. Artes, R.; Barroso, L. P. Métodos multivariados de análise estatística. São Paulo: Blucher, 2023. 534 p.
5. Shahapure, K. R.; Nicholas, C. Cluster Quality Analysis Using Silhouette Score. 2020 IEEE 7th International Conference on Data Science and Advanced Analytics (DSAA), Sydney, NSW, Australia, 2020, pp. 747-748, doi: 10.1109/DSAA49011.2020.00096.
6. R Core Team. R: A Language and Environment for Statistical Computing. R Foundation for Statistical Computing, Vienna, Austria, 2024. <https://www.R-project.org/>. (accessed 2024–08–31).
